# Rapamycin rescues loss of function in blood-brain barrier–interacting Tregs

**DOI:** 10.1172/jci.insight.167457

**Published:** 2024-02-22

**Authors:** Paulien Baeten, Ibrahim Hamad, Cindy Hoeks, Michael Hiltensperger, Bart Van Wijmeersch, Veronica Popescu, Lilian Aly, Veerle Somers, Thomas Korn, Markus Kleinewietfeld, Niels Hellings, Bieke Broux

**Affiliations:** 1 Universitair MS Centrum, Campus Diepenbeek, Belgium.; 2 Department of Immunology and Infection, Biomedical Research Institute, Hasselt University, Diepenbeek, Belgium.; 3 VIB Laboratory of Translational Immunomodulation, Center for Inflammation Research (IRC), Department of Immunology and Infection, Biomedical Research Institute, Hasselt University, Diepenbeek, Belgium.; 4 Klinikum Rechts der Isar, Institute for Experimental Neuroimmunology, Technische Universität München, Munich, Germany.; 5 Universitair MS Centrum, Campus Pelt, Belgium.; 6 Noorderhart, Revalidatie & MS Centrum, Pelt, Belgium.; 7 Munich Cluster for Systems Neurology (SyNergy), Munich, Germany.

**Keywords:** Autoimmunity, Immunology, Cell migration/adhesion, Multiple sclerosis, Tolerance

## Abstract

In autoimmunity, FOXP3^+^ Tregs skew toward a proinflammatory, nonsuppressive phenotype and are, therefore, unable to control the exaggerated autoimmune response. This largely affects the success of autologous Treg therapy, which is currently under investigation for autoimmune diseases, including multiple sclerosis (MS). There is a need to ensure in vivo Treg stability before successful application of Treg therapy. Using genetic fate-mapping mice, we demonstrate that inflammatory, cytokine-expressing exFOXP3 T cells accumulate in the CNS during experimental autoimmune encephalomyelitis. In a human in vitro model, we discovered that interaction with inflamed blood-brain barrier endothelial cells (BBB-ECs) induces loss of function by Tregs. Transcriptome and cytokine analysis revealed that in vitro migrated Tregs have disrupted regenerative potential and a proinflammatory Th1/17 signature, and they upregulate the mTORC1 signaling pathway. In vitro treatment of migrated human Tregs with the clinically approved mTORC1 inhibitor rapamycin restored suppression. Finally, flow cytometric analysis indicated an enrichment of inflammatory, less-suppressive CD49d^+^ Tregs in the cerebrospinal fluid of people with MS. In summary, interaction with BBB-ECs is sufficient to affect Treg function, and transmigration triggers an additive proinflammatory phenotype switch. These insights help improve the efficacy of autologous Treg therapy of MS.

## Abstract 2

Transmigration across an inflamed blood-brain barrier affects human Treg stability, which can be reversed by rapamycin treatment.

## Graphical abstract

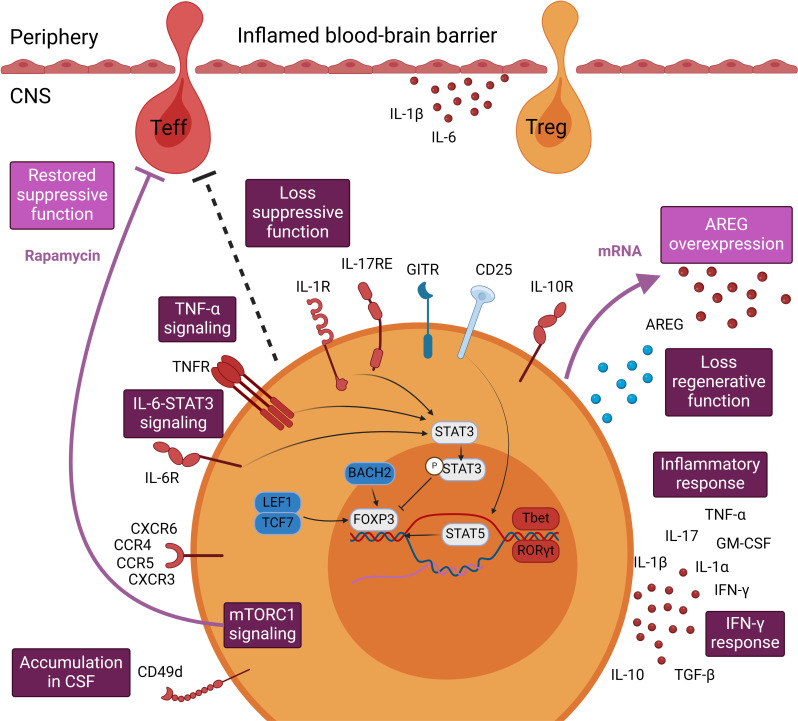

## Introduction

Reestablishing tolerance holds the most potential for treating autoimmunity. More than a decade ago, researchers started to investigate the body’s own immunosuppressive machinery, resulting in clinical trials using Treg-based cell therapies (1). Although reported to be safe, efficacy is largely lacking (2, 3). This failure could be due to the described loss of Treg phenotype in autoimmunity (4–6), as we and others showed for multiple sclerosis (MS) (7–9). MS is a chronic demyelinating disorder of the CNS, mediated by an autoimmune reaction. After an initiating event (potentially Epstein-Barr virus infection; ref. 10) in a genetically susceptible individual, peripherally activated autoreactive CD4^+^ Th cells initiate blood-brain barrier (BBB) disruption, causing influx of circulating inflammatory immune cells into the brain (11–13). This exaggerated local immune response leads to demyelination and axonal loss in white and gray matter (14). As a result, loss of vision, memory, and sensation and even paralysis can occur. Current treatments mainly focus on relieving inflammation and, thus, delaying disease progression, while therapies targeting demyelination and neurodegeneration are missing. Therefore, new therapies avoiding general immunosuppression and inducing remyelination and neuroregeneration are needed. Interestingly, FOXP3^+^CD4^+^CD25^hi^CD127^lo^ Tregs not only control immune responses but also induce regeneration in the CNS via amphiregulin (AREG) and CCN3 (15–18). Therefore, Treg-based therapies may provide a promising therapeutic option for MS.

The BBB limits access of soluble factors and immune cells from the blood into the brain. This specialized function is mediated by the expression of intercellular tight junctions and specific transporters on BBB-endothelial cells (BBB-ECs), as well as the glia limitans produced by astrocytes (19). In MS, BBB integrity is affected and expression of chemokines and adhesion molecules is induced, thereby favoring immune cell entry into the perivascular space and, ultimately, the brain parenchyma (20). Although Tregs intrinsically have an increased migratory capacity compared with effector T cells (Teff), this function is impaired in people with relapsing-remitting MS (RRMS) (21). Nonetheless, Tregs are found in the cerebrospinal fluid (CSF) of people with RRMS (8, 9, 22) and in active brain lesions (22). In experimental autoimmune encephalomyelitis (EAE), Tregs found in the CNS are unable to suppress CNS-resident Teff. This effect is mediated by IL-6 and TNF-α produced by Teff (23). Also, IFN-γ–producing Tregs with downregulated FOXP3 expression (exFOXP3 T cells) were found in the CNS of EAE animals (24). Interestingly, it was shown that monocytes migrating through the BBB differentiate into DCs, which promote inflammatory Th17 cell polarization (25). Indeed, human Tregs also skew toward proinflammatory Th1- and Th17-like cells in autoimmunity, depending on the microenvironment (26, 27). This raises the question of whether Tregs are affected by BBB transmigration in a neuroinflammatory setting as well. In the context of Treg therapy, this in vivo Treg instability needs to be addressed to ensure safe and successful treatment (2, 3).

We investigated the phenotype and function of Tregs interacting with an inflamed BBB. We demonstrate that inflammatory, cytokine-expressing exFOXP3 T cells accumulate in the CNS of FOXP3 fate-mapping mice during the course of EAE. In a human in vitro BBB model, we found that suppression by Tregs interacting with inflamed BBB-ECs was affected, in contrast to interaction with control BBB-ECs. Further analysis on the transcriptome of migrated Tregs from healthy donors (HD) and people with untreated RRMS (uRRMS) was performed. We show that Th1/17 pathways and mammalian target of rapamycin complex 1 (mTORC1) signaling are upregulated in human BBB-transmigrated Tregs compared with nonmigrated Tregs. In uRRMS-derived Tregs specifically, Th17-related pathways were increased after migration, suggesting a preexisting susceptibility to Th17 skewing of Tregs in people with MS. In addition, downregulation of AREG in migrated Tregs suggests a loss of regenerative capacity, which can be restored using mRNA technology. These findings were corroborated in EAE mice, where CNS-derived exFOXP3 T cells display similar changes. Treatment of human migrated Tregs with the mTORC1 inhibitor rapamycin restored Treg function. Importantly, uRRMS-derived Tregs were still sensitive to rapamycin. Finally, an enrichment of inflammatory, less-suppressive CD49d^+^ Tregs was found in the CSF of people with uRRMS, thereby underscoring the relevance of our findings for human disease.

Our results show that interaction with inflamed BBB-ECs induces loss of function in Tregs and that transmigration induces an additive proinflammatory skewing, which should be addressed before applying Treg therapy to people with autoimmune diseases such as MS. Interfering in any of the identified pathways, as illustrated by the use of rapamycin, has the capacity to restore and maintain Treg phenotype for the development of a stable cell therapy.

## Results

### Loss of phenotype and function in BBB-transmigrated Tregs.

**Figure 1:**
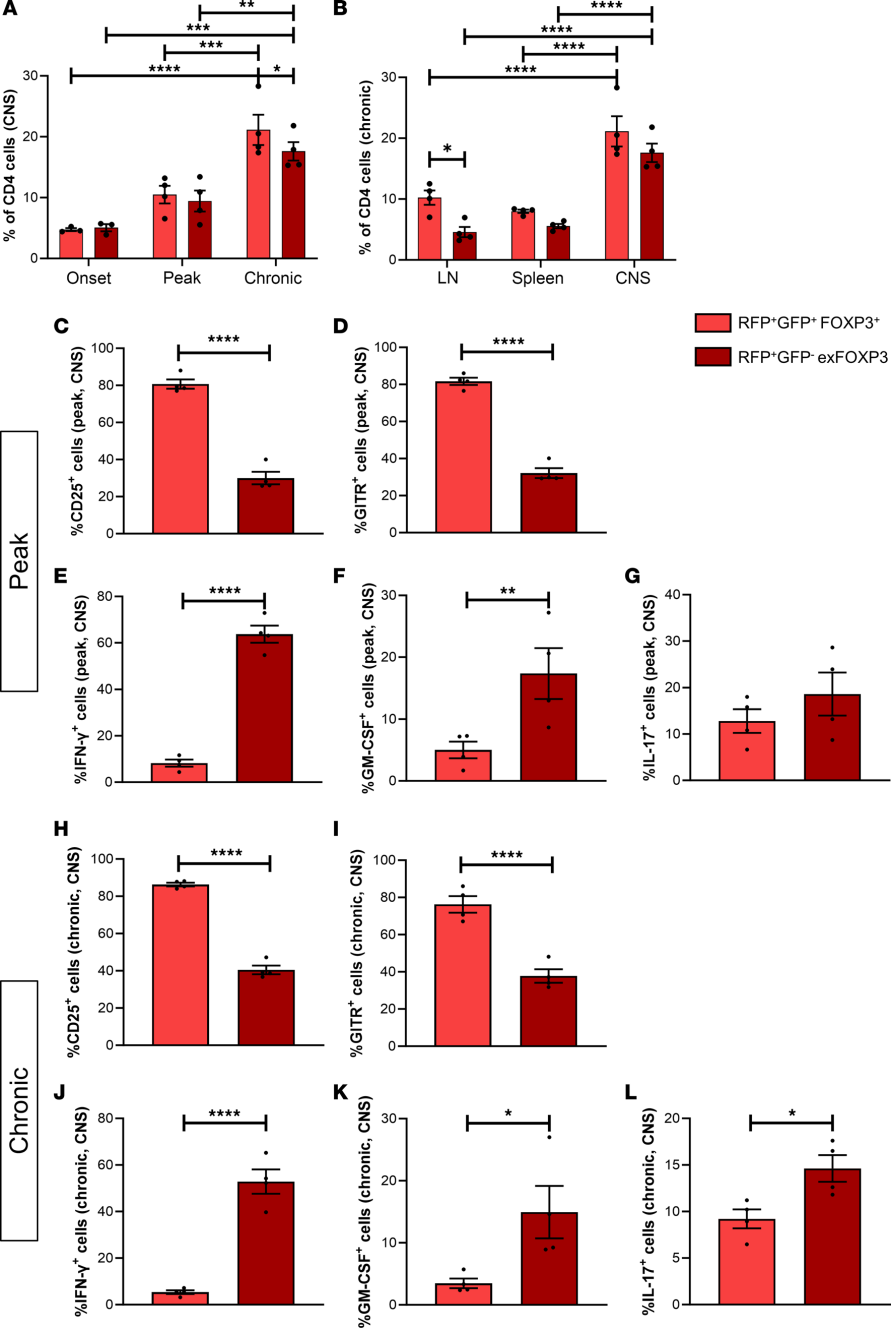
Inflammatory exFOXP3 T cells are enriched in the inflamed CNS in vivo. EAE was induced in female FOXP3^Cre-GFP^ Rosa^RFP^ fate-mapping mice. Tissues were collected at onset, peak, or chronic phase of disease (represented by the arrows in Supplemental Figure 1), and immune cells were isolated from spleen, lymph nodes (LN), or pooled brain and spinal cord (CNS) for flow cytometry. (**A**) Percentage of FOXP3^+^ Tregs and exFOXP3 T cells in the CNS along EAE course. (**B**) Percentage of FOXP3^+^ Tregs and exFOXP3 T cells at the chronic phase in different tissues. (**C**–**L**) Isolated cells at peak or chronic phase were stimulated for 4 hours with PMA, ionomycin, and Golgiplug. Percentage of CD25^+^ (**C** and **H**), GITR^+^ (**D** and **I**), IFN-γ^+^ (**E** and **J**), GM-CSF^+^ (**F** and **K**), and IL-17^+^ (**G** and **L**) within either FOXP3^+^ Tregs or exFOXP3 T cells in CNS. Representative dot plot of RFP versus GFP in splenocytes and gating strategy in Supplemental Figure 1. *n* = 3–5; 2-way ANOVA with Bonferroni’s multiple-comparison test. Data are plotted as mean ± SEM. *P < 0.05; **P < 0.01; ***P < 0.001; ****P < 0.0001.

Studies in mice have shown that the CNS is enriched for Tregs that are proinflammatory and nonsuppressive (23, 24). Since it was previously shown that migration across the BBB affects the phenotype of monocytes (25), we investigated whether the BBB is involved in the reported Treg phenotype switch and loss of function. To interrogate FOXP3 expression of Tregs after migration into the CNS, we induced active EAE by myelin oligodendrocyte glycoprotein (MOG_35-55_) immunization in FOXP3^Cre-GFP^ Rosa^RFP^ fate-mapping mice. Here, GFP and Cre-recombinase were introduced into the genome downstream of the FOXP3 promotor. Following FOXP3 expression, GFP and Cre-recombinase are transcribed, resulting in FOXP3^+^GFP^+^ cells. In addition, the Cre-recombinase removes a floxed STOP codon upstream of RFP, both downstream of the Rosa26 promotor. As a consequence of the continuous transcription of Rosa26, RFP will be continuously expressed, independently of FOXP3 expression, whereas GFP expression is dependent of FOXP3 expression. This transgenic model results in a Treg-specific expression of GFP and RFP, enabling discrimination of bona fide FOXP3^+^ Tregs (RFP^+^GFP^+^) from exFOXP3 T cells (RFP^+^GFP^–^) using flow cytometry (Figure 1 and Supplemental Figures 1 and 2; supplemental material available online with this article; https://doi.org/10.1172/jci.insight.167457DS1). We found that exFOXP3 T cells accumulate in the CNS over time (Figure 1A). In the chronic phase of EAE, exFOXP3 T cells were significantly increased in the CNS compared with the periphery (Figure 1B). FOXP3^+^ Tregs outnumber the proportion of exFOXP3 T cells in lymph nodes, while both cell populations are found in equal proportions in the CNS. Both populations were further phenotyped for Treg markers and inflammatory cytokines (Figure 1, C–L, and Supplemental Figure 2). Frequency of CD25^+^ (Figure 1, C and H) and GITR^+^ (Figure 1, D and I) cells were significantly decreased in exFOXP3 T cells compared with FOXP3^+^ Tregs of the CNS during peak and chronic phase of EAE. ExFOXP3 T cells show a proinflammatory phenotype including increased frequency of IFN-γ^+^ (Figure 1, E and J), GM-CSF^+^ (Figure 1, F and K), and IL-17^+^ (Figure 1, G and L) cells. Strikingly, the percentage of IL-17–expressing FOXP3^+^ Tregs is also significantly increased in the CNS compared with the periphery (Supplemental Figure 2, E and J). This suggests that loss of FOXP3 is not required for IL-17 production. Altogether, these results show that exFOXP3 T cells are enriched in the inflamed CNS in vivo, coinciding with loss of Treg markers and gain of an inflammatory profile.

**Figure 2:**
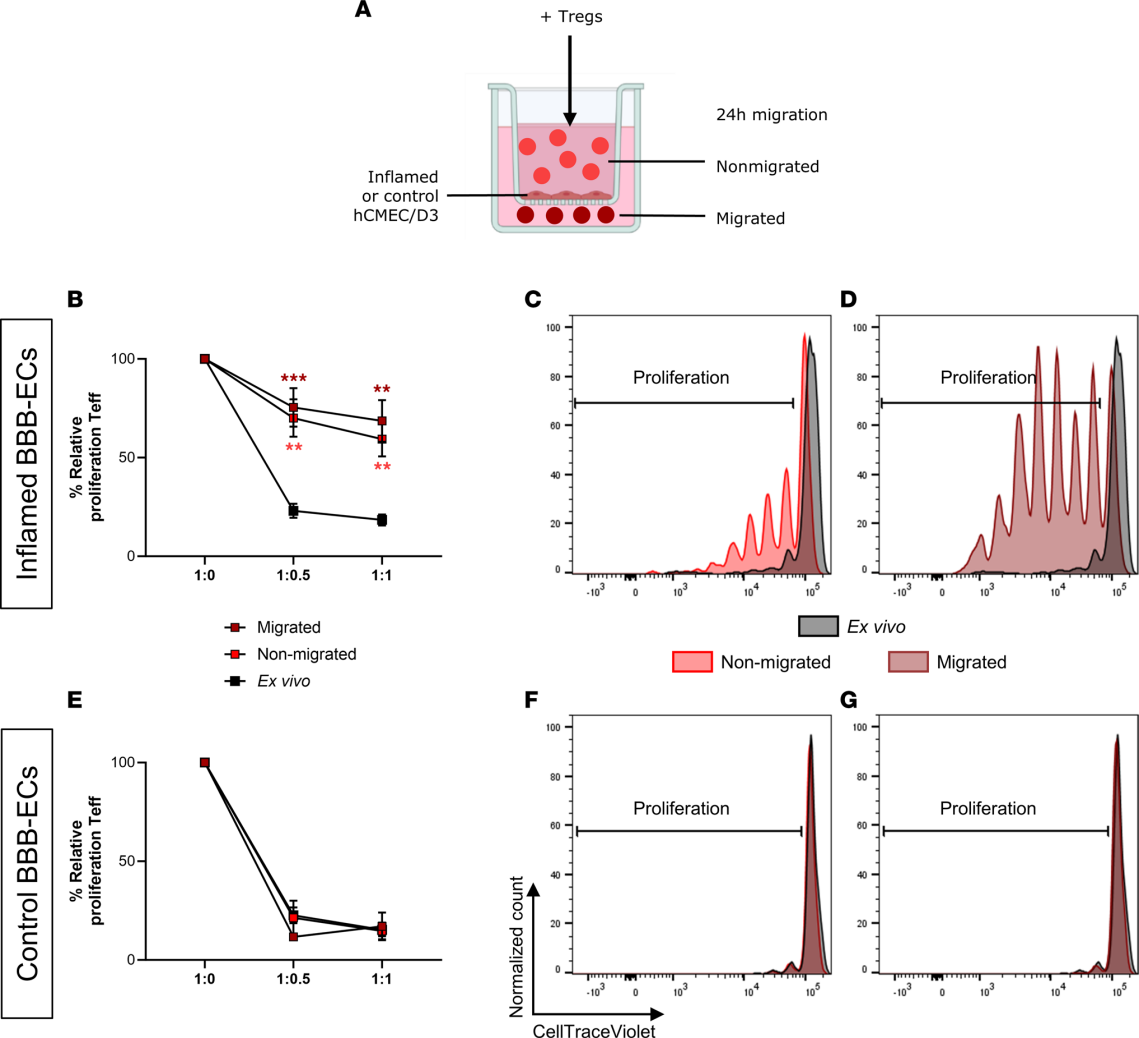
Interaction with inflamed but not control BBB-ECs causes loss of function in human Tregs. (**A**) Schematic representation of Boyden chamber migration assay. hCMEC/D3 cells were stimulated with or without (control) IFN-γ and TNF-α (inflamed) for 24 hours followed by washing. Next, human Tregs were sorted by FACS from HD-derived PBMCs and used immediately as ex vivo Tregs or loaded on the chamber for migration assay. After 24 hours, migrated and nonmigrated Tregs were collected for further analysis. (**B**–**G**) Suppressive capacity of ex vivo Tregs, nonmigrated Tregs, or migrated Tregs after interaction with inflamed (**B**) or control (**E**) BBB-ECs. Representative dilution plots of CellTraceViolet of ex vivo and nonmigrated (**C** and **F**; 1:1) or ex vivo and migrated (**D** and **G**; 1:1) Tregs. Percentage proliferation represents CellTrace dilution of Teff in suppression assays with different ratios of Teff and Tregs (given as Teff/Treg). Relative proliferation is normalized to 1:0 condition (100%). Dilutions plots are normalized to the maximum count of the represented conditions. Gating strategy in Supplemental Figure 4. *n* = 4–8; 2-way ANOVA with Bonferroni’s multiple-comparison test compared with ex vivo condition per ratio. **P < 0.01; ***P < 0.001.

To identify whether BBB-ECs are responsible for Treg instability, we used the human brain EC line hCMEC/D3 in a modified Boyden chamber migration assay to mimic the BBB in vitro (Figure 2A) (28). This model enables us to look at the direct effect of control or inflamed BBB-ECs on Treg function and phenotype. Tregs were allowed to migrate across BBB-ECs, and the suppressive capacity of migrated and nonmigrated Tregs was determined. After contact with inflamed BBB-ECs (Figure 2, B–D), both nonmigrated and migrated Tregs displayed a reduced suppressive capacity compared with ex vivo Tregs. In contrast, suppressive capacity was not affected by interaction with control BBB-ECs (Figure 2, E–G). These results suggest that interaction with inflamed BBB-ECs is sufficient to induce a loss of function in Tregs.

### BBB transmigration activates inflammatory and mTORC1 pathways in human and mouse Tregs.

**Figure 3:**
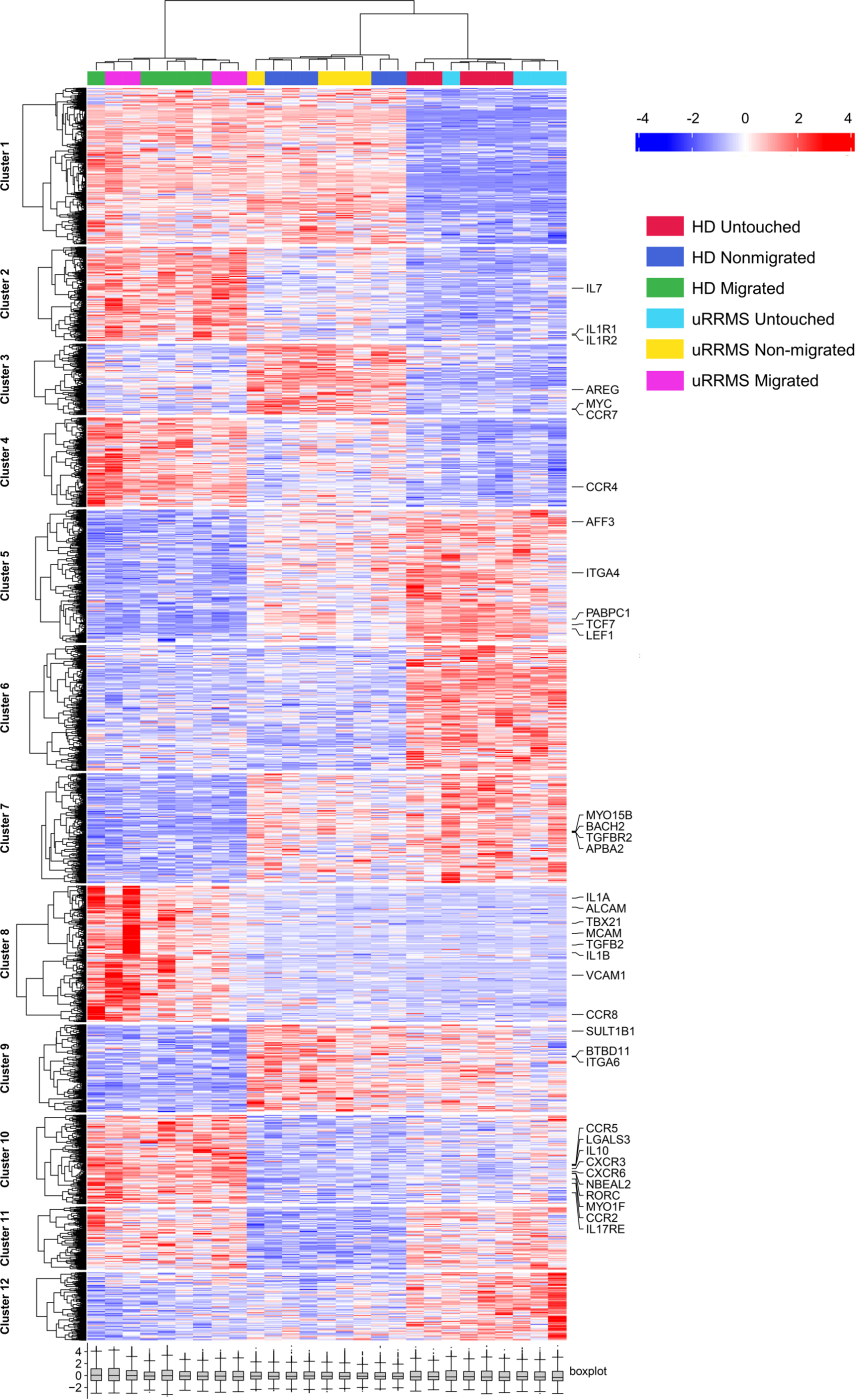
Human BBB-EC–transmigrated Tregs present a dysfunctional, proinflammatory phenotype. HD- and uRRMS-derived Tregs were loaded on an inflamed Boyden chamber migration assay as represented in Figure 2A. Tregs were also cultured alone for 24 hours in the same EC medium (untouched Tregs). After 24 hours, untouched, migrated, and nonmigrated Tregs were collected and bulk RNA-Seq was performed. Hierarchical clustering showing changes in gene expression of the experimental conditions. Relative gene expression is indicated by color: upregulation in red and downregulation in blue. Genes and samples with similar expression were automatically grouped (left and top trees). Inflammation-, migration-, and regulation-related genes are highlighted. Expression values are shown as *Z* scores. Benjamini-Hochberg adjusted *P* value (FDR < 0.05) was used to determine DEGs. *n* = 5 (HD) and *n* = 4 (uRRMS).

**Figure 4:**
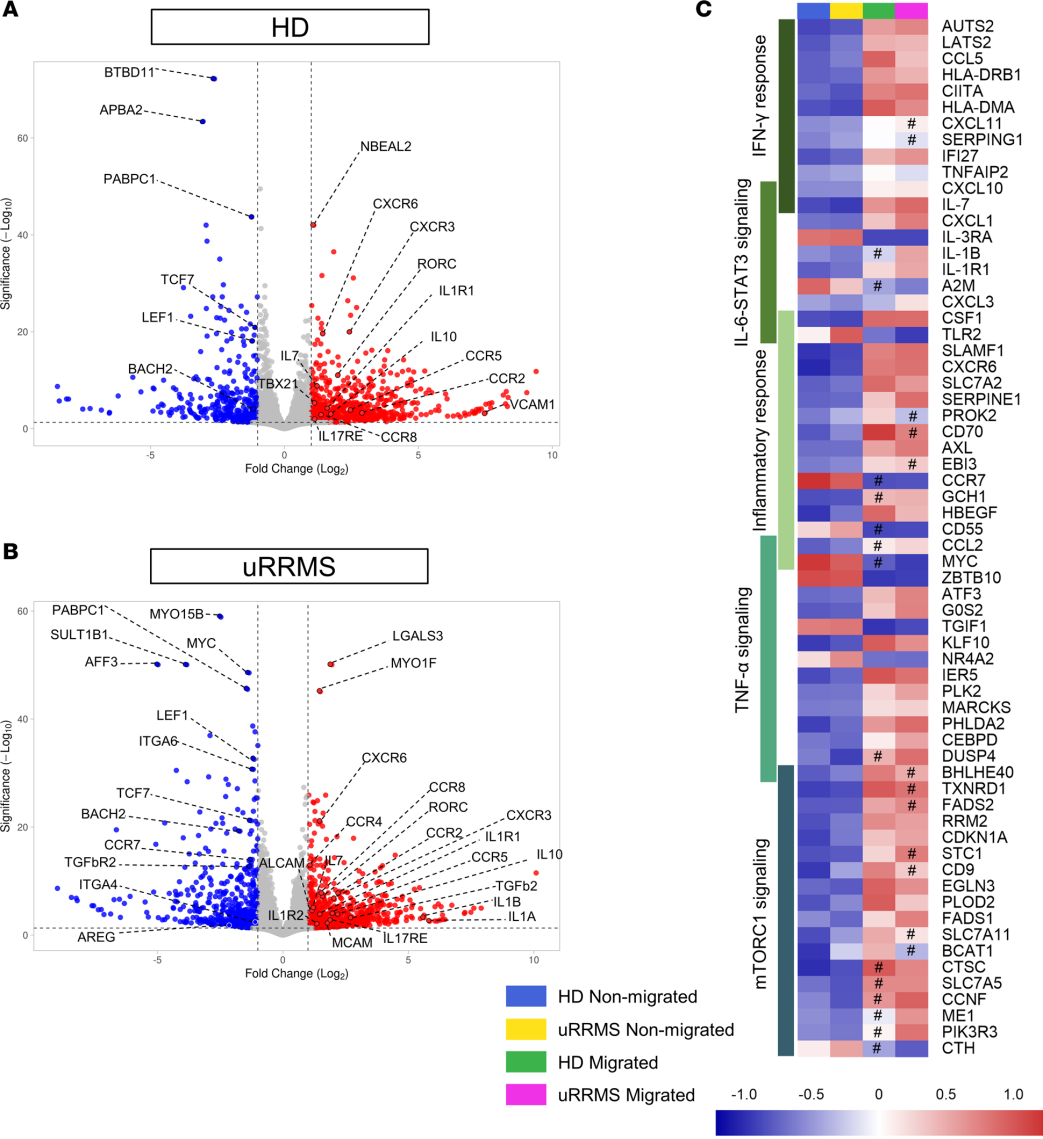
Pathway analysis shows upregulation of inflammatory phenotype and mTORC1 signaling in migrated Tregs. Bulk RNA-Seq was performed on untouched, migrated, and nonmigrated Tregs. (**A** and **B**) Volcano plot of DEGs in migrated versus nonmigrated Tregs for HD (**A**) and uRRMS (**B**). The *x* axis shows the log_2_ fold change for ratio migrated/nonmigrated. The *y* axis shows statistical significance (FDR-adjusted *P* value). Up- and downregulated genes are colored red or blue, respectively, if adjusted *P* < 0.05 and |log_2_ fold change| > 1. Inflammation-, migration-, and regulation-related genes are highlighted. (**C**) The 10 most significant DEGs were grouped into following pathways (GSEA identified; Supplemental Table 2, and Supplemental Figure 5): inflammatory response, TNF-α signaling, mTORC1 signaling, IFN-γ response, and IL-6-STAT3 signaling (only enriched in migrated uRRMS-derived Tregs). Relative gene expression is indicated by color: upregulation in red and downregulation in blue. Expression values are given as *Z* scores; Benjamini-Hochberg adjusted *P* value (FDR < 0.05) and |log_2_ fold change| > 1; nonsignificant differences (FDR > 0.05 or |log_2_ fold change| > 1) are marked with #. *n* = 5 (HD) and *n* = 4 (uRRMS)

To reveal transcriptional changes in BBB-transmigrated human Tregs, RNA-Seq was performed on untouched, migrated, and nonmigrated Tregs from 5 HD and 4 persons with uRRMS (Figures 3 and 4). Clustering analysis shows that untouched Tregs (no contact with BBB-ECs, cultured for 24 hours in EC medium) from HD and uRRMS cluster separately from migrated and nonmigrated Tregs (Figure 3). Specifically, gene cluster 1 is downregulated, while clusters 6 and 12 are upregulated in untouched Tregs compared with the BBB-interacting Tregs. To identify the changes induced by contact with BBB-ECs, genes shared between migrated and nonmigrated Tregs were compared with genes in untouched Tregs. This analysis revealed that genes related to the JAK3/STAT pathway (29–34) were upregulated in BBB-interacting Tregs (Supplemental Figure 3); this could explain the loss of function identified in Figure 2, since JAK/STAT signaling is known to regulate FOXP3 expression (35) and since JAK3 is involved in IL-2– and IL-10–mediated downstream signaling (36). Finally, additional transcriptional changes were found in migrated Tregs compared with nonmigrated Tregs. Clusters 2, 8, and 10 are upregulated in migrated Tregs, while clusters 7 and 9 are downregulated compared with nonmigrated Tregs. Gene annotation thereof reveals an increased inflammatory and decreased Treg phenotype. Together with the in vivo data showing accumulation of exFOXP3 T cells in the CNS, this highlights the importance of identifying differences between migrating and nonmigrating (but nonetheless interacting) Tregs.

Next, we investigated specific transcriptional changes related to BBB transmigration. Significant differences in gene expression between nonmigrated and migrated Tregs in HD and persons with uRRMS were analyzed (Figure 4, A and B). We found an increased expression of inflammation-related (e.g., *TNFRSF8/9*, *TBX21*, *RORC*, *IL17RE*, *IL1*, *IL1R*) and migration-related (e.g., *VCAM1*, *ALCAM*, *MCAM*, *CXCR3*, *CCR4*, *CXCR6*, *CCR5*) differentially expressed genes (DEGs), while Treg genes (e.g., *TGFbR2*, *LEF1*, *TCF7*, *BACH2)* were downregulated. Of importance, *AREG* was downregulated only in MS-derived migrated Tregs, suggesting loss of regenerative capacity (Figure 4B).

Ingenuity pathway analysis (IPA) and gene set enrichment analysis (GSEA) (top 20 pathways in Supplemental Tables 1 and 2 and most applicable pathways in Supplemental Figure 5) were performed to investigate relevant molecular pathways affected in migrated Tregs compared with nonmigrated Tregs. As expected, the actin cytoskeleton signaling pathway was increased; this is related to the transmigration process. Moreover, several inflammation-related pathways were affected by BBB transmigration of Tregs. These include the inflammatory and neuroinflammatory response, mTORC1 signaling, IFN-γ response (Th1 pathway) and TNF-α signaling for both HD and uRRMS, and IL-6-STAT3 signaling (IL-17 pathway) exclusively for uRRMS (Figure 4C, Supplemental Figure 5, and Supplemental Tables 1 and 2). Together with the loss of *AREG*, these results indicate that migrated Tregs from people with uRRMS differ from HD-derived migrated Tregs, identifying disease-specific pathological features. In summary, we show that BBB-transmigrated Tregs display an additive proinflammatory phenotype switch on top of loss of function due to interaction with BBB-ECs, resembling the Treg phenotype frequently observed in persons with autoimmunity (26, 27).

**Figure 5:**
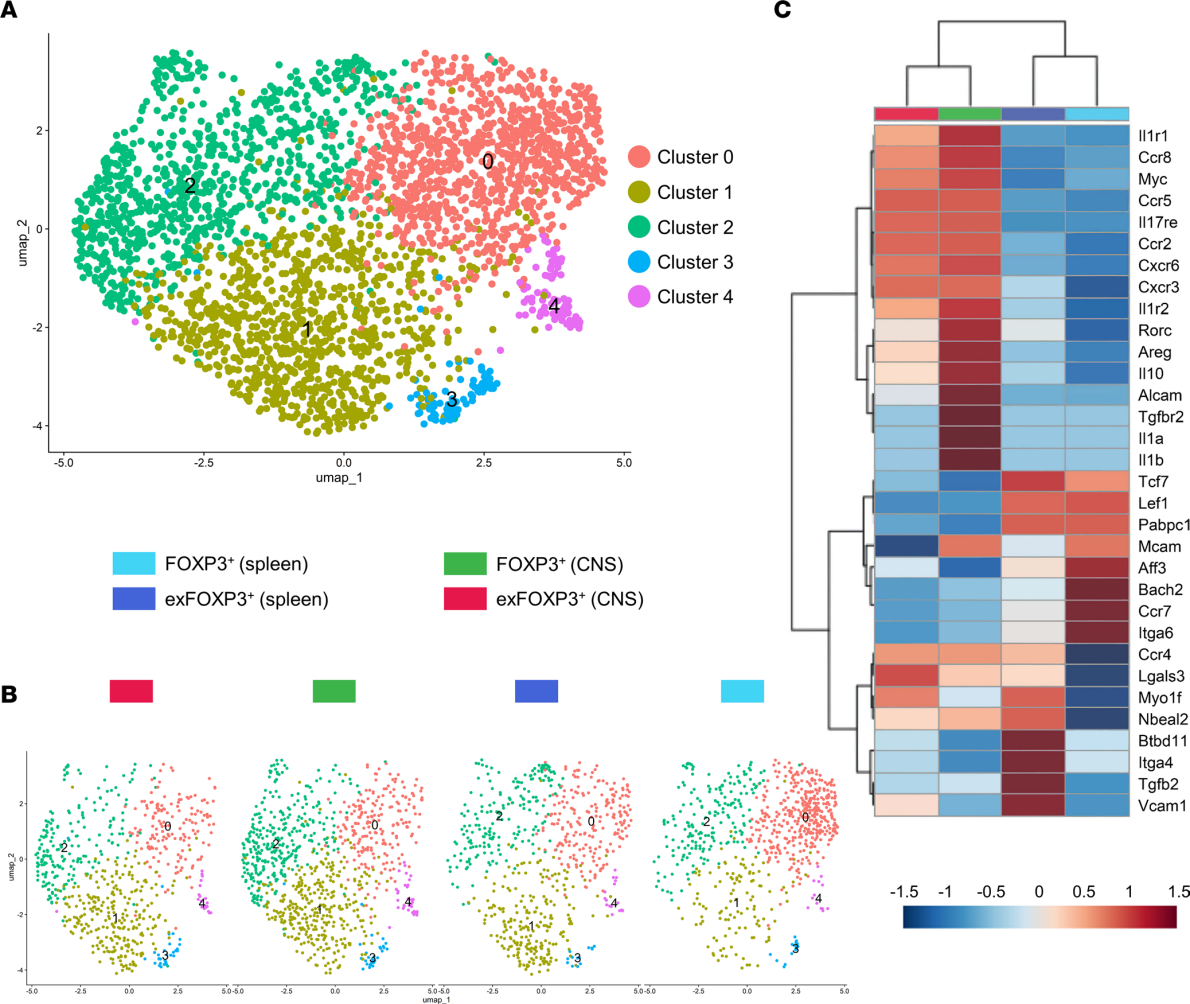
Inflammatory genes are upregulated in CNS-isolated Tregs. EAE was induced in female FOXP3^Cre-GFP^ Rosa^RFP^ fate-mapping mice. Tissues were collected at peak (18 dpi; Supplemental Figure 1), and RFP^+^GFP^+^ FOXP3^+^ Tregs and RFP^+^GFP^–^ exFOXP3 T cells were sorted by FACS from spleen and pooled brain and spinal cord (CNS). Gating strategy in Supplemental Figure 1. Single-cell RNA-Seq was performed on CNS- and spleen-derived RFP^+^GFP^–^ exFOXP3 T cells and RFP^+^GFP^+^FOXP3^+^ Tregs. (**A** and **B**) Two-dimensional UMAP plot showing the clustering of 3,029 cells based on gene expression divided into 5 clusters. Point coordinates are based on UMAP dimensionality reduction of the top 15 principal components. Individual points correspond to single cells colored according to clusters (**A**) and samples (**B**). Sample representation in UMAP and top expressing genes per cluster in Supplemental Figure 6. (**C**) Hierarchical clustering showing changes in gene expression of the experimental conditions. Relative gene expression is indicated by color: upregulation in red and downregulation in blue. Genes and samples with similar expression were automatically grouped (left and top trees). Inflammation-, migration-, and regulation-related genes are highlighted. Expression values are shown as *Z* scores. Differential expression was analyzed using the Wilcoxon ranked-sum test.

To validate our findings in vivo, we compared gene expression of RFP^+^GFP^+^ (FOXP3^+^) Tregs with RFP^+^GFP^–^ exFOXP3 T cells from the spleen and CNS of mice at peak of EAE by single-cell RNA-Seq (Supplemental Figure 1). Clustering analysis revealed 5 distinct Treg populations (Figure 5 and Supplemental Figure 6), which were cross-referenced to the subset signatures as identified by Miragaia et al. (37). Cluster 0 (*Ccr7*, *Bcl2*, *Bach2*, *Tcf7*) is mostly defined by splenic FOXP3^+^ Treg, and corresponds to central Tregs (cTregs). Cluster 1 (*Gzmb*, *Ccr4*, *Lgals3*, *Ccr2*) mostly consists of splenic exFOXP T cells and CNS-derived cells, and it corresponds to nonlymphoid tissue (NLT) Tregs. Cluster 2 (*Itgb8*, *Il10*, *Nkg7*, *Lag3*) is found in all samples and correspond to suppressive Tregs. Cluster 3 (*Ifit3b*, *Mx1*, *Cmpk2*) and Cluster 4 (*Il17a*, *Tnfsf11*, *Il1r1*, *Klrc1*) are mostly composed of CNS-derived cells, irrespective of the Treg subpopulation. These populations are characterized by inflammatory signatures mixed with a NLT phenotype. For IL-17, these data are in line with our flow cytometry results, showing that the frequency of IL-17^+^ cells is not different during EAE peak but only during the chronic phase (Figure 1). Interestingly, the transcriptional pattern of murine CNS-derived exFOXP3 T cells is highly similar to that of human BBB-transmigrated Tregs, with comparable regulation of genes like *Il1r1*, *Il1r2*, *Il17re*, *Ccr2*, *Ccr5*, *Ccr8*, *Cxcr3*, and *Cxcr6* (Figure 5C). However, many other inflammation-related genes (*Il1b*, *Il1a*, *Roc*) show highest expression in FOXP3^+^ Tregs of the CNS. Treg-related genes *Tcf7*, *Lef1*, and *Bach2* are mostly expressed in splenic FOXP3^+^ Tregs (cTreg) and are decreased in CNS-derived Tregs, even in FOXP3^+^ Tregs. This is in line with our previous in vivo results (Figure 1), suggesting that FOXP3-expressing Tregs in the CNS acquire a proinflammatory phenotype while losing their Treg signature. However, some differences between CNS-derived Tregs and exFOXP3 T cells could be identified, including downregulation of *Areg*, *Il10*, *Tgfb2*, *Alcam*, *Mcam*, *Il1a*, and *Il1b* and upregulation of *Vcam1* and *Lgals3* in exFOXP3 T cells compared with Tregs. These data show that the proinflammatory skewing of BBB-transmigrating Tregs in vitro mimics in vivo results.

In summary, we show that migration across BBB-ECs induces an altered phenotype in Tregs in vitro and in vivo, as highlighted by the upregulated proinflammatory Th1 and mTORC1 signaling pathways in migrated Tregs. The Th17-related IL-6/STAT3 signaling pathway and the regenerative gene *AREG* are uniquely affected in MS-derived migrated Tregs, identifying disease-specific pathological features. Finally, we confirmed this proinflammatory phenotype in CNS-isolated Tregs.

### Migrated Tregs show a migratory, inflammatory phenotype with loss of regenerative phenotype.

**Figure 6:**
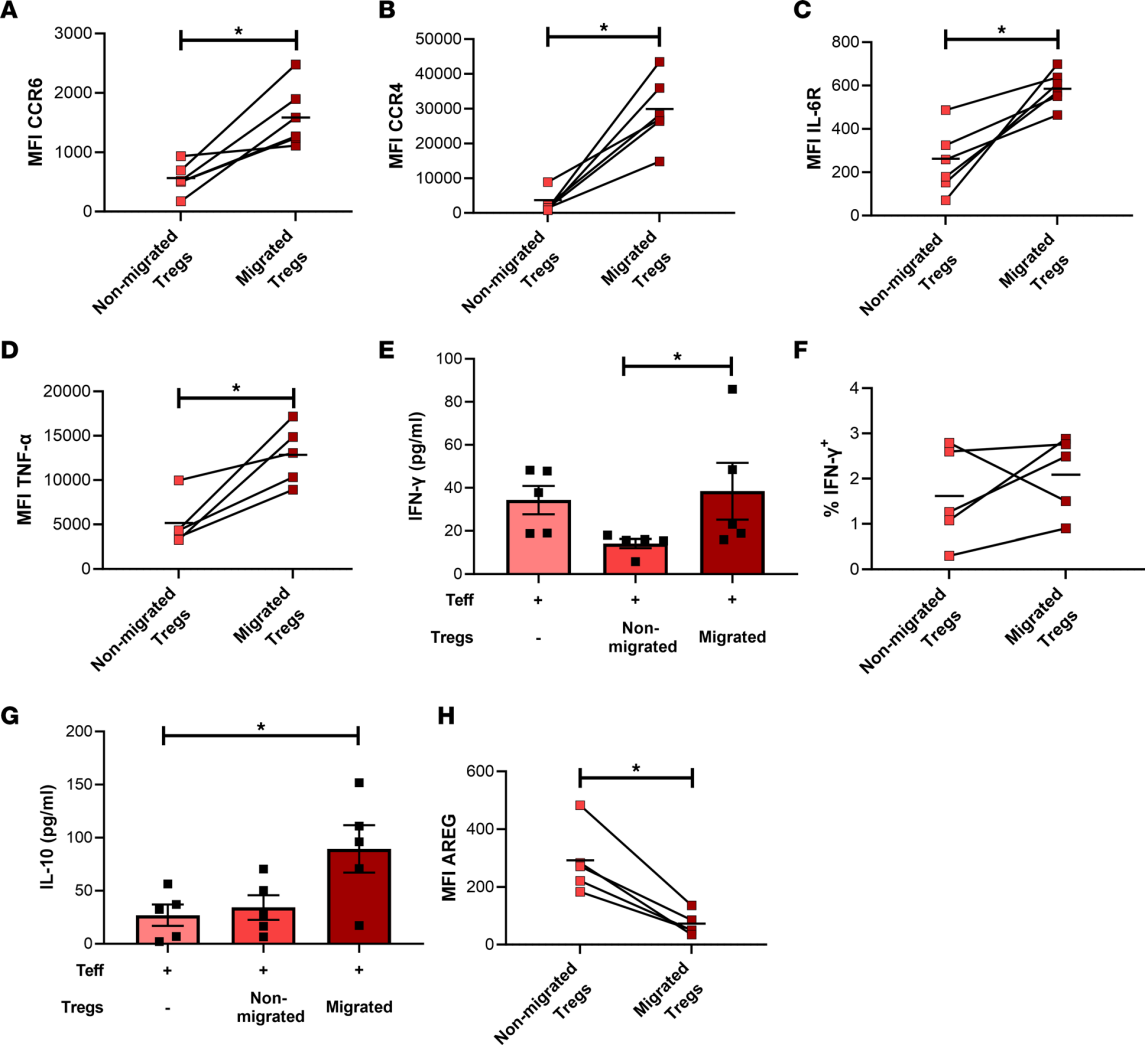
Proinflammatory phenotype of migrated human Tregs on protein levels. HD-derived migrated and nonmigrated Tregs were collected and either directly phenotyped or phenotyped after coculture with Teff. (**A**–**D**, **F**, and **H**) Using flow cytometry, molecules identified by pathway analysis were validated. MFI of CCR6 (**A**), MFI of CCR4 (**B**), MFI of IL-6R (**C**), MFI of TNF-α (**D**), percentage of IFN-γ^+^ cells (**F**), or MFI of AREG (**H**) of live cells are shown. Horizontal bars represent group mean. *n* = 5–6; One-tailed Wilcoxon test. Gating in Supplemental Figure 9. (**E** and **G**) Concentration of IFN-γ (**E**) and IL-10 (**G**) were measured in supernatants of Teff/Treg cocultures (1:0 or 1:1). *n* = 5; Friedman test with Dunn’s multiple-comparison test. *P < 0.05

Flow cytometry, bead-based immunoassays and ELISA were employed to validate selected targets and interesting pathways from the human RNA-Seq experiment at a protein/functional level. CCR6 and CCR4 were increased on migrated Tregs, in line with the identified migratory, inflammatory phenotype (Figure 6, A and B). The Th17-inducing molecule IL-6R (38) was increased on migrated Tregs, confirming pathway analysis (Figure 6C). In line, we found that inflamed BBB-ECs produce high levels of IL-6 and IL-1β at the time of coculture with Tregs (Supplemental Figure 7), revealing a potential mechanism for Th17 skewing of Tregs. Next, using flow cytometry and bead-based immunoassays, TNF-α (Figure 6D), IFN-γ (Figure 6, E and F), and IL-10 (Figure 6G) expression or cytokine production by migrated and nonmigrated Tregs was measured, respectively. In line with the RNA-Seq analysis, TNF-α expression is significantly increased in migrated Tregs compared with nonmigrated Tregs (Figure 6D). Cocultures of Teff with migrated Tregs resulted in higher IFN-γ levels in the supernatant compared with Teff cocultured with nonmigrated Tregs (Figure 6E). However, direct analysis of the Treg subpopulations show low percentages of IFN-γ^+^ cells with no difference due to migration (Figure 6F). Thus, the data suggest that the increased IFN-γ expression is derived from Teff that are no longer suppressed by Tregs. In line with our RNA-Seq data, IL-10 production is also higher in cocultures when migrated Tregs are used, compared with Teff alone and also compared with coculture with nonmigrated Tregs (Figure 6G). However, this is not sufficient to override the loss of function in migrated Tregs. Lastly, our RNA-Seq data show a decreased expression of *AREG* by migrated Tregs from people with uRRMS. Here, we validated this loss of AREG protein expression in migrated Tregs from HD as well (Figure 6H). In addition, no AREG could be detected in the CSF of uRRMS by ELISA, and plasma concentrations were very low (2.384 ± 0.9364 pg/mL; mean ± SEM). Therefore, we investigated whether we can boost AREG expression in primary human Tregs. Using mRNA transfection, AREG expression could be induced in human Tregs, reaching a frequency of 91.68% ± 2.546% (mean ± SEM) AREG-expressing Tregs (Supplemental Figure 8A). The percentage of AREG^+^ cells dropped to 15.6% ± 5.825% (mean ± SEM) after 48 hours and decreased rapidly after 72 hours. Although expression was lost beyond 4 days after transfection, the secreted AREG in the supernatant remains stable for 4 days (Supplemental Figure 8B). This exhibits the feasibility and potential of ex vivo mRNA transfection in primary human Tregs, thereby enhancing the therapeutic potential of Treg cell therapy.

In summary, key proteins related to migration, inflammation, and regeneration were validated on migrated Tregs using flow cytometry, bead-based immunoassays, and ELISA, confirming their proinflammatory phenotype and highlighting the potential of targeting any of the identified key targets to enhance therapeutic potential.

### Rapamycin treatment restores the suppressive capacity of migrated Tregs.

**Figure 7:**
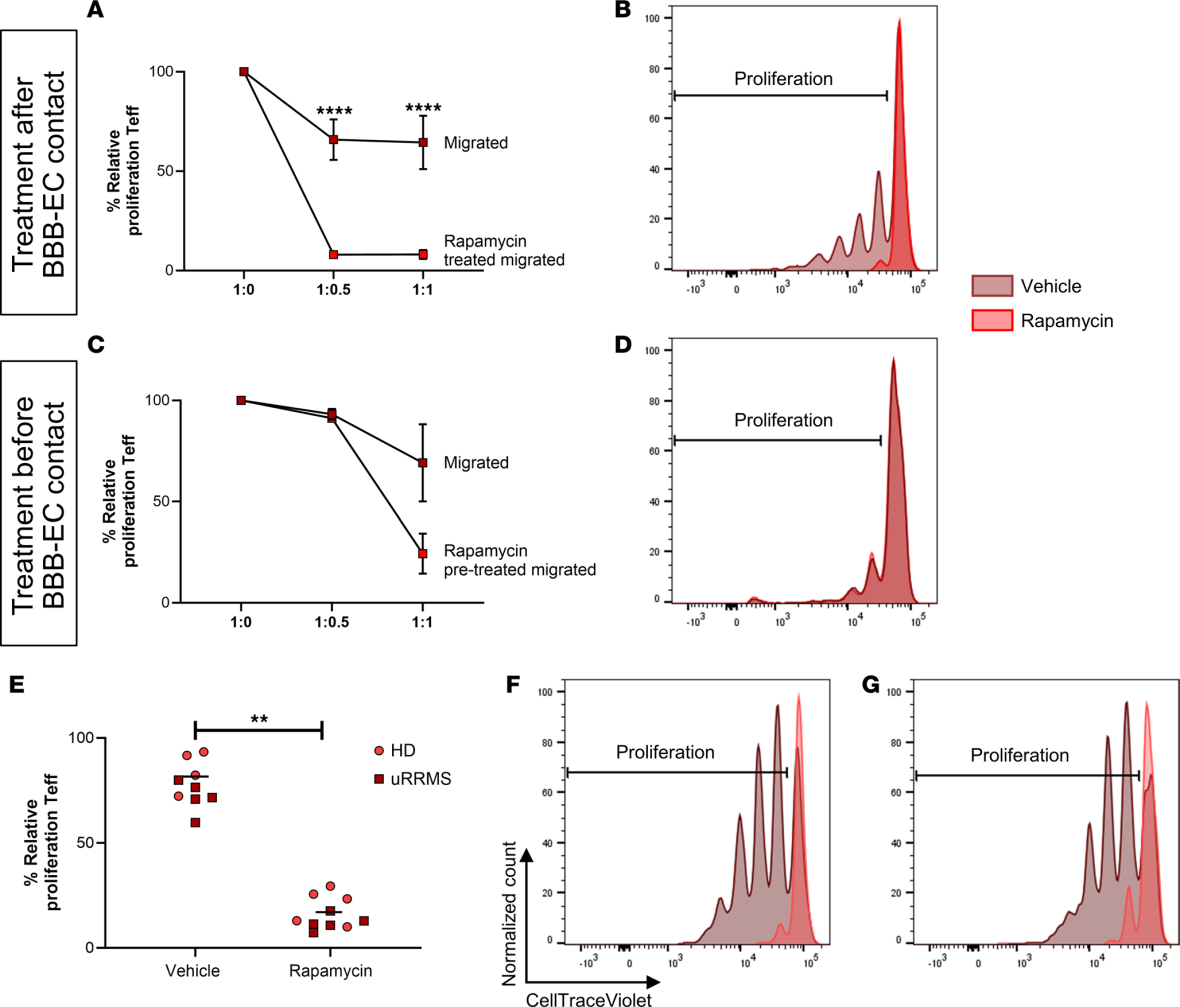
Loss of suppressive capacity of migrated human Tregs is restored by rapamycin. (**A** and **B**) Migrated HD-derived Tregs were treated with rapamycin (2 μM) for 4 hours, washed, and cocultured with Teff in a suppression assay. Showing representative plots of migrated Tregs with and without (vehicle) rapamycin treatment (**B**; 1:1). (**C** and **D**) HD-derived Tregs were treated with rapamycin (2 μM) for 4 hours and put on the Boyden chamber migration assay. After 24 hours, migrated Tregs were collected and cocultured with Teff. Showing representative plots of migrated Tregs with and without (vehicle) rapamycin treatment (**D**; 1:1). Percentage proliferation represents CellTrace dilution of Teff. Cell ratio is given as Teff/Treg. Relative proliferation is normalized to 1:0 condition (100%). Gating in Supplemental Figure 3. *n* = 3–6; 2-way ANOVA with Bonferroni’s multiple-comparison test. (**E**–**G**) Frozen PBMCs of HD and people with uRRMS were thawed, and Tregs were sorted by FACS. Immediately after isolation, Tregs were treated with rapamycin (2 μM) or vehicle for 4 hours, washed, and cocultured with Teff in a suppression assay (1:1). Showing representative plots of migrated Tregs with and without rapamycin treatment of HD (**F**) and persons with uRRMS (**G**). Horizontal bars represent group mean. *n* = 5 (HD), *n* = 5 (uRRMS); Wilcoxon test. **P < 0.01; ****P < 0.0001.

One of the most prominently affected Treg function–related pathways by transmigration, is the mTORC1 signaling pathway. This pathway promotes Th differentiation while inhibiting Treg induction (39). Especially Th17 differentiation via reduced IL-6–induced STAT3 phosphorylation is shown to be lost with mTORC1 inhibition (40). The mTORC1 inhibitor rapamycin is already being used in preclinical MS models (41, 42) and in clinical trials (NCT01903473; refs. 43, 44). To determine whether inhibition of mTORC1 restores Treg function after BBB transmigration, migrated Tregs were pretreated with rapamycin before coculturing with Teff. Interestingly, rapamycin restored and even augmented the suppressive capacity of migrated Tregs (Figure 7, A and B; nonmigrated Tregs in Supplemental Figure 10). In contrast, treating Tregs with rapamycin before BBB transmigration did not result in a stable protection of Treg function (Figure 7, C and D, and Supplemental Figure 10). To test whether Tregs from persons with uRRMS are still sensitive to rapamycin-enhanced suppressive capacity, Tregs were sorted from frozen PBMCs. Immediately after isolation, Tregs were pretreated with rapamycin before coculturing with Teff. Indeed, these results show that resting Tregs of people with uRRMS are equally sensitive to rapamycin as HD-derived Tregs (Figure 7, E–G). Our data demonstrate that rapamycin restores the loss of function of migrated human Tregs, highlighting the potential of targeting any of the identified pathways to maintain Treg stability in the context of MS.

### Proinflammatory and nonsuppressive Tregs accumulate in CSF of people with MS.

**Figure 8:**
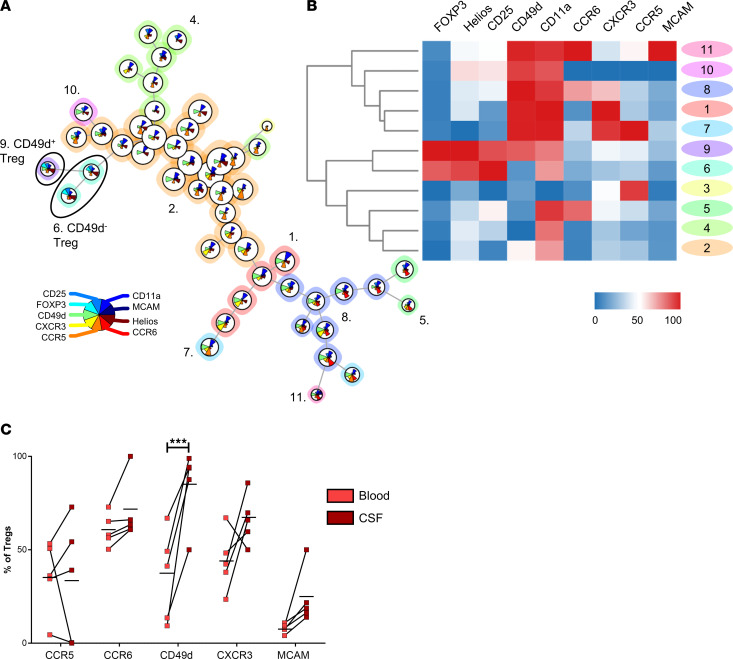
Proinflammatory CD49d^+^ Tregs accumulate in the CSF of people with MS. (**A** and **B**) Frozen PBMCs of HD and people with different MS types were thawed and studied using flow cytometry (gating in Supplemental Figure 11). (**A**) FlowSOM analysis on CD4 T cells identified 2 subpopulations of Tregs. (**B**) Heatmap related to the FlowSOM analysis. Color scale as percentiles. *n* = 26 (HD), *n* = 24 (uRRMS), *n* = 25 (flRRMS), *n* = 23 (uSPMS). (**C**) Fresh, paired blood, and CSF samples were collected from persons with uRRMS at diagnosis and studied using flow cytometry. Percentage expression of different migratory molecules within single, live, CD4^+^CD25^hi^CD127^lo^ Tregs illustrated that CD49d^+^ Tregs are enriched in the CSF. Gating in Supplemental Figure 12. Horizontal bars represent mean of the group. *n* = 5; 2-way ANOVA with Bonferroni’s multiple-comparison test. ***P < 0.001.

Having established that Tregs lose their phenotype and suppressive function after migration across inflamed BBB-ECs in vitro and in vivo, we sought evidence to evaluate this phenotype switch in people with MS. Blood samples of 26 HD, 24 people with uRRMS, 25 people with first-line–treated RRMS (flRRMS), and 23 people with untreated secondary-progressive MS (uSPMS) were analyzed by flow cytometry. Pooled data were analyzed using FlowSOM, which uses self-organizing maps for visualization of flow cytometry data in an unbiased way (45). We identified the presence of 2 Treg subpopulations in the peripheral blood, which are distinguished by CD49d expression (Figure 8, A and B, cluster 6 and 9). CD49d was previously found to be a marker for inflammatory and nonsuppressive Tregs (46), although in this cohort, its expression is not different between blood of HD and people with MS (Supplemental Figure 11). In contrast, analysis of paired blood and CSF samples of persons with uRRMS at time of diagnosis revealed that CD49d^+^ Tregs (single, live, CD4^+^CD25^hi^CD127^lo^) were significantly enriched in the CSF (Figure 8C and Supplemental Figure 12). In line with our previous results, these data indicate that inflammatory and less-suppressive Tregs are not yet expanded in the blood of people with MS; however, they do accumulate in the CSF.

## Discussion

The in vivo stability of Tregs is crucial for the success of Treg-based cell therapy in autoimmunity (2, 3). The identification of Treg instability at the site of inflammation, and its driving factors, will enable us to improve Treg therapy efficacy and safety. Loss of FOXP3 expression has been shown to occur in Tregs in inflammatory microenvironments, resulting in exFOXP3, nonsuppressive cells (23, 24, 26). Because of the autoreactive nature of Tregs, conversion to Teff poses a major risk for using Treg therapy. Here, we investigated the role of Treg-BBB interaction and transmigration on the function and phenotype of Tregs in MS. We found that, in a mouse model for MS, exFOXP3 cells accumulate in the CNS during the course of EAE. These exFOXP3 T cells had lost Treg markers while expressing inflammatory cytokines GM-CSF, IL-17, and IFN-γ. Using a human in vitro model, we provide evidence for a human Treg population with an altered phenotype and lost suppressive function after interaction with inflamed BBB-ECs. Even more, Tregs migrating across inflamed BBB-ECs display a proinflammatory phenotype, which is most pronounced in people with uRRMS. Selective migration of a specific Treg subset cannot be ruled out; however, nonmigrated Tregs, representing interacting yet non–EC-adherent cells without transmigration, represent loss of function and a transcriptome already divergent from untouched Tregs as well. Even more, the JAK/STAT pathway was upregulated in Tregs that interacted with BBB-ECs compared with untouched Tregs, suggesting a direct effect of the inflamed BBB-ECs on the observed loss of function (35, 36). Subsequently, transcriptomic analysis of migrated Tregs revealed that several inflammatory pathways (IFN-γ response, TNF-α signaling, and IL-6-STAT3 pathway) and the mTORC1 signaling were upregulated compared with nonmigrated Tregs. These pathways were validated on protein level, as demonstrated by the increased IL-6R expression and TNF-α expression by migrated Tregs. Although not sufficient to override the reduced suppressive function, migrated Tregs and exFOXP3 T cells from the CNS show increased IL-10 and *Tgfb2* expression, which could indicate a compensation mechanism; however, autocrine TGF-β production by Th17 cells was also shown to maintain Th17 cells (47), and it is known to be important for Th17 differentiation (48). Furthermore, the regenerative potential of Tregs, illustrated by the expression of AREG, is disrupted in migrated Tregs as well, which could be reversed by mRNA-induced overexpression. When using MS-derived Tregs in the migration assay, we found unique upregulation of the Th17 signaling pathway, identifying disease-specific pathological features. These results were validated in FOXP3^+^ Tregs and exFOXP3 T cells from EAE mice. FOXP3^+^ Tregs isolated from the spleen mostly express genes related to the cTreg signature (*Ccr7*, *Bach2*, *Tcf7*, *Lef1*), while exFOXP3 T cells from the CNS most express key genes related to inflammation (*Il17a*, *Tnfsf11*, *Il1r1*) and migration to tissues. Treatment of migrated Tregs with mTORC1 inhibitor rapamycin fully restored their suppressive capacity, even in Tregs from people with uRRMS. These results provide proof of principle that interfering in any of the identified pathways has the capacity to stabilize Treg phenotype. Finally, we show that less-suppressive and inflammatory Tregs, previously characterized by expression of CD49d (46), accumulated in the CSF of persons with MS, supporting our human in vitro and murine in vivo results. These CD49d^+^ Tregs were not yet expanded in the blood of people with MS but did accumulate in the CSF of persons with MS. However, we cannot rule out that this effect does not result from selective migration of CD49^+^ Tregs.

Previous studies identified nonsuppressive and proinflammatory Tregs in the CNS of EAE mice, which thereby contribute to disease pathogenesis (23, 24). These CNS-resident Tregs were previously shown to produce IFN-γ and were unable to suppress CNS-isolated Teff. Using a FOXP3^Cre-GFP^ Rosa^RFP^ fate-mapping mouse model, we now show that exFOXP3 T cells accumulate in the CNS along the EAE disease course. Using this model, one cannot rule out that this enrichment is not due to clonal expansion; however, due to low IL-2 levels in the CNS, this might be unlikely (49). Further experiments will need to clarify these results. We demonstrate loss of function and phenotype in human Tregs after contact with and migration across inflamed BBB-ECs in vitro, and we confirmed key inflammatory-related DEGs in CNS-derived exFOXP3 T cells and FOXP3^+^ Tregs in vivo in the fate-mapping mouse model. These data suggest that, although FOXP3^+^ Tregs are present in the CNS during EAE, they represent a proinflammatory phenotype.

The BBB consists of multiple cellular players, but we show in our in vitro unicellular model that inflamed BBB-ECs are sufficient to induce significant changes in migrating Tregs. Indeed, this is in line with the report of Ifergan et al. (25), who showed that migration of monocytes across BBB-ECs induced skewing toward Th17-differentiating DCs. Even more, contact alone is sufficient to decrease the suppressive capacity of Tregs and induce transcriptomic changes, which are further aggravated by transmigration, suggesting a true alteration and no selective expansion. The conversion of migrating immune cells is suggested to be driven solely by factors expressed or produced by BBB-ECs, like IL-6. We confirmed production of IL-6 and IL-1β by inflamed hCMEC/D3 cells at the time of coculture, while we showed that IL-6R and *IL-1R* expression are increased on migrated Tregs. However, no functional correlations were yet determined and further research is necessary. Indeed, literature has shown IL-6R expression on human Tregs as well (50), which renders Tregs — and more specifically thymic Tregs (51) — more susceptible to Th17 skewing (52, 53). Previous reports show that IL-1 and IL-6 alone and together downregulate FOXP3 while inducing IL-17 expression on Tregs in a STAT3-dependent way (38, 40, 54–57); this is also apparent in EAE and rheumatoid arthritis (23, 26, 58). Since these cytokines are not BBB specific, these effects will not be limited to MS. Indeed, fibroblast-produced IL-6 also drives conversion of Tregs into IL-17–producing exFOXP3 cells accumulating in the inflamed joints of mice with autoimmune arthritis (26). Tocilizumab, an IL-6R blocking antibody, is already approved for treatment of rheumatoid arthritis (59, 60) and has been successfully applied to hospitalized people with COVID-19 (61), showing its potential in dampening hyperinflammatory responses. In addition, blocking IL-1R in human Tregs was shown to prevent IL-17 production in vitro (55). The marketed drug IL-1R antagonist Anakinra is currently under investigation in clinical trials for MS (NCT04025554; ref. 62). These findings highlight the importance of the STAT3 signaling pathway in Th17 differentiation, and we here identified an MS-specific upregulation of this pathway in migrated Tregs. Further studies are needed to evaluate the effect of IL-6 and/or IL-1β blocking antibodies in preventing the detrimental inflammatory effects of BBB-ECs on migrating Tregs.

Kim et al. (40) showed that, in the context of hepatitis B liver failure, the STAT3-based Th17/Treg balance can be regulated by mTORC1 inhibitor rapamycin. Rapamycin is shown to alleviate EAE symptoms and related Th1- and Th17-mediated inflammation while boosting Treg numbers (41, 42). Although rapamycin plays an important role in Th cells (39), mTORC1 inhibition is here performed on in vitro Tregs only. Indeed, rapamycin was previously found to selectively expand highly suppressive Tregs in vitro, to maintain Treg function and phenotype, and to prevent a pathogenic switch under Th skewing conditions (63–65). Therefore, rapamycin is already being used in the context of MS and Treg cell therapy (2, 3, 43, 44). Our in vitro RNA-Seq results show that the mTORC1 signaling is activated in migrated Tregs showing a proinflammatory phenotype and lost suppressive function. Altogether, this prompted us to use rapamycin in our experiments, with the goal of rescuing the altered function of migrated Tregs. Indeed, we found that treatment of migrated Tregs with rapamycin recovered and even improved their suppressive capacity. Even more, Tregs from people with uRRMS were still susceptible to rapamycin. This reveals the potential of targeting any of the identified mechanisms in this set up to stabilize Treg phenotype in the context of Treg-based cell therapy for autoimmunity. However, treatment of Tregs with rapamycin before BBB transmigration did not prevent loss of function. In the context of Treg therapy, this suggests that a transient treatment of ex vivo Tregs before infusion would not be sufficient to maintain in vivo stability, which is crucial for effective Treg therapy in MS. Rather, genetically interfering in any of the described pathways holds the potential to result in a stable Treg-based cell therapy for persons with MS.

Next to loss of their regulatory function, our results show that the regenerative capacity of Tregs is altered, as well, after BBB-transmigration. We demonstrate a reduction of AREG expression on migrated Tregs on both mRNA and protein levels. Since Tregs have been reported to induce regeneration in the CNS using AREG (17), this suggests that migrated Tregs lose this ability. Therefore, we aimed to enhance AREG expression by Tregs using mRNA transfection, which led to a strong AREG induction stable up to 4 days. However, the regenerative capacity of MS-derived Tregs has not been studied yet, although this is of high interest since regenerative therapies are currently lacking for people with MS.

Altogether, these results indicate that interaction with BBB-ECs and transmigration is detrimental for human Tregs in vitro and in vivo. Interaction with BBB-ECs is sufficient to affect Treg function, and transmigration triggers an additive proinflammatory phenotype switch that is most pronounced in people with uRRMS. Interestingly, CNS-derived exFOXP3 T cells of EAE mice were transcriptionally highly similar to human BBB-transmigrating Tregs. Finally, we found that CD49d^+^ Tregs accumulate in the CSF of recently diagnosed persons with uRRMS, thereby confirming our human in vitro and murine in vivo results. Altogether, the relevance of our findings was validated in the first clinical trial using Treg therapy in MS; Chwojnicki et al. showed that intrathecally administrated Tregs show more clinical potential compared with i.v. administered Tregs (66). This finding supports our results and could explain disease progression in people with MS receiving i.v. infusion of Tregs. However, due to low patient recruitment in this phase I trial, these conclusions should be approached with caution. In order to move forward with Treg-based cell therapies for autoimmunity, permanently overcoming and even preventing the observed phenotype switch will likely result in a stable and safe cellular product for clinical use. Our research identifies several pathways that can be used to restore Treg function, as we show with rapamycin as a proof of principle, to ensure Treg stability, even in highly inflammatory conditions like the MS brain environment.

## Methods

### Sex as a biological variable.

Our study exclusively examined female mice because the disease model is only relevant in females. Both male and female participants with different types of MS or healthy donors were included in our study.

### Animals.

BAC-FOXP3^Cre-GFP^ mice (67, 68) and Rosa26^fl^STOP^fl^RFP mice (69), with a C57BL/6 background, were crossed to obtain BAC-FOXP3^Cre-GFP^ Rosa26^fl^STOP^fl^RFP (FOXP3^Cre-GFP^ Rosa^RFP^) fate-mapping mice, enabling the discrimination of RFP^+^GFP^+^FOXP3^+^ Tregs and RFP^+^GFP^–^ exFOXP3 T cells in vivo. Animals were housed in an accredited conventional animal facility under a 12-hour light/dark cycle and had free access to food and water.

### EAE induction.

Ten-week-old female mice were s.c. injected with MOG_35-55_ emulsified in Complete Freund’s Adjuvant containing *Mycobacterium tuberculosis*, according to manufacturer’s instructions (Hooke Laboratories). Immediately after immunization and on day 1, mice were i.p. injected with 100 ng/100 μL pertussis toxin. Animals were weighed daily, and neurological deficits were evaluated using a standard 5-point scale (0, no symptoms; 1, limp tail; 2, weakness of hind legs; 3, complete paralysis of hind legs; 4, complete hind and partial front leg paralysis; 5, death). Analysis of the clinical EAE scores was performed using pooled data from 3 independent experiments. At EAE onset (12 days postinduction [dpi]), EAE peak (14 or 18 dpi), and the chronic phase of disease (28 dpi) lymph nodes, spleen, spinal cord, and brains were isolated after transcardial perfusion with Ringer’s solution. A single-cell suspension from lymph nodes and spleen was derived by mechanical transfer through a 70 μm cell strainer (Greiner Bio-One). For the CNS, both enzymatic digestion, using collagenase D (Roche Diagnostics GmbH) and DNase I (Roche Diagnostics GmbH), and mechanical dissociation were performed, followed by a Percoll gradient (GE Healthcare). Isolated cells were stimulated with phorbol 12-myristate 13-acetate (PMA, Merck), ionomycin (Merck), and Golgiplug (BD Biosciences) for 4 hours at 37°C at 5%CO_2_. Next, cells were stained with Zombie NIR or Zombie Aqua, CD4 Pacific Blue (RM4-5) or APC-Fire750 (RM4-5), GITR PerCP/Cy5.5 (DTA-1), CD25 APC (PC61), GM-CSF PE/Cy7 (MP1-22E9), IFN-γ BV605 (XMG1.2), and IL-17 BV421 (TC11-18H10.1; all from BioLegend) and were acquired on BD LSRFortessa (BD Biosciences) and analyzed using FlowJo 10.8.0 (BD Biosciences). In addition, cells isolated from spleen and CNS were stained with Zombie NIR and sorted using the FACSAria Fusion (BD Biosciences) for RFP^+^GFP^+^ FOXP3^+^ Tregs and RFP^+^GFP^–^ exFOXP3 T cells. The gating and sorting strategy is depicted in Supplemental Figure 1, and purity of all sorts was confirmed to be > 90%.

### Single-cell RNA-Seq.

Sorted RFP^+^GFP^+^FOXP3^+^ Tregs and RFP^+^GFP^–^ exFOXP3 T cells isolated from spleen and CNS from EAE mice were stained with sample tag antibodies (Ms Single Cell Sampling Multiplexing Kit, BD Biosciences). Next, cells were pooled and immediately used for single-cell capture using the BD Rhapsody Express system (BD Biosciences). Directly following cell capture, DNA library preparation was performed using the BD Rhapsody WTA Amplification Kit (BD Biosciences) according to manufacturer’s instructions. In short, after single-cell capture in the nanowell plate, Cell Capture beads were loaded and cells were lysed. Free-floating mRNA was allowed to bind to the Cell Capture bead present in the same nanowell, and beads were collected. Next, reverse transcription and exonuclease I treatment were performed. Prior to mRNA amplification, Sample Tag products were denatured off the Cell Capture beads. WTA and Sample Tag libraries were prepared in parallel. After each amplification round and following index-PCR, the PCR products were purified using Agencourt AMPure XP beads (Beckman Coulter Diagnostics). Concentration of final PCR products were determined using dsDNA High Sensitivity assay kit (Thermo Fisher Scientific) on the Qubit analyzer (Thermo Fisher Scientific). Quality check was performed with the Bioanalyzer 2100 using the Agilent High Sensitivity DNA Kit (both from Agilent). Pooled sequencing-ready libraries of the WTA and Sample Tag were sequenced on Illumina NovaSeq 6000 (150 bp, paired end reads) at Novogene Co Ltd. Following service provider instructions, Seven Bridges Genomics was used to perform quality control, to map reads against mm10 using STAR, and to determine expression counts. The transcript count matrix was loaded into R (v4.2.3) using the Seurat (v3.0) package (70). The number of genes was log normalized, and the 2,000 most variably expressed genes were extracted using a variance-stabilizing transformation implemented in the FindVariableFeatures function in Seurat. Principal Component Analysis was then performed using this pruned gene list. Uniform Manifold Approximation and Projection (UMAP) was performed based on the first 15 PCs using the implementation in the python package umap-learn with correlation as the distance measure in PC space. UMAP were created on both 2- and 3-dimensional spaces. To define cell clusters, a shared nearest neighbor (SNN) graph was created using Seurat:FindNeighbors based on the first 15 PCs, and the cell clusters were defined on the SNN graph using Seurat:FindClusters. Differential expression was analyzed using the Wilcoxon ranked-sum test included in Seurat:FindMarkers.

### Patients.

Peripheral blood samples were obtained from HD and people with MS and were collected by Noorderhart Hospital (Pelt, Belgium) and the University Biobank Limburg (UBiLim, Hasselt, Belgium) (71). Buffy coats of HD were purchased from the Belgian Red Cross. Samples were used immediately or stored in liquid nitrogen. Paired CSF and blood samples were collected from people with MS at diagnosis in the Klinikum Rechts der Isar, Technical University of Munich (Munich, Germany). Donor characteristics are given in Supplemental Table 3.

### Treg isolation.

Tregs were isolated from peripheral blood mononuclear cells (PBMCs) by negative selection of CD4 (Miltenyi Biotec) followed by positive CD25 selection (Miltenyi Biotec) using magnetic beads according to manufacturer instructions. Next, Tregs were sorted using a FACSAriaII or FACSAria Fusion (BD Biosciences) using the following antibodies: CD4 APC-eFluor780 (OKT4) and CD127 PE (eBioRDR5; both from Thermo Fisher Scientific) as well as CD25 PE/Cy7 (M-A251), CD25 Kiravia Blue 520 (M-A251), CD127 BV421 (A019D5), CD127 PE/Cy5 (A019D5), CD4 AF700 (RPA-T4), and CD4 BV421 (RPA-T4; all from BioLegend). The sorting strategy is depicted in Supplemental Figure 13, and purity of all sorts was confirmed to be > 95%. Tregs (CD4^+^CD25^hi^CD127^lo^) and Teff (CD4^+^CD25^–^) were used in the Boyden chamber migration assay and the suppression assay.

### hCMEC/D3 cell culture.

The human brain EC line hCMEC/D3 was obtained at Tebubio. Cells were used up to passage 35. Cells were cultured in collagen coated (rat tail, type I, Sigma-Aldrich) culture flasks in growth medium (EGM-2MV medium [Lonza] supplemented with 2.5% FBS [Thermo Fisher Scientific]). Cells were collected using trypsin and cultured in 24-well plates or in Thincerts (both Greiner Bio-One) for migration assays as described further. After reaching near confluency (90%), cells were replenished with experimental medium (basal EBM2 [Lonza] supplemented with Gentamicin [10 μg/mL], Amphotericin B [1 μg/mL], fibroblast growth factor [FGF, 1 ng/mL], hydrocortisone [HC, 1.4 μM; all Sigma-Aldrich], 2.5% FBS [Thermo Fisher Scientific]). One day later, cells were treated with TNF-α (100 ng/mL) and IFN-γ (10 ng/mL, both from Peprotech) (72) for 24 hours in reduced medium (basal EBM2 [Lonza] supplemented with Gentamicin, Amphotericin, FGF, 0.25% FBS) (73).

### Boyden chamber migration assay.

For migration assays, the Boyden chamber migration assay was used as described before (28, 73). hCMEC/D3 were trypsinized and cultured in collagen-coated Thincerts (24-well, translucent, 3 μm, Greiner Bio-One) at a density of 25 × 10^3^ cells/cm^2^. On days 3 and 5, cells were replenished with experimental medium as described before. Growth of the monolayer was followed using measurement of transepithelial electrical resistance (TEER) using EVOM2 Epithelial Voltohmmeter (World precision instruments) and reached a plateau phase on day 6. Then, cells were treated with and without (control) TNF-α (100 ng/mL) and IFN-γ (10 ng/mL, all Peprotech) for 24hin reduced medium as described before. Before adding Tregs, the inserts were washed and put in a new plate with fresh reduced medium. (Pretreated) Tregs (4 × 10^5^ to 1 × 10^6^) were allowed to migrate or were cultured in reduced medium as a control condition (untouched) for 24 hours. Tregs from the well (migrated) and from the insert (nonmigrated) were collected for RNA isolation, flow cytometry, or further in vitro culturing. For RNA isolation, Tregs were pelleted and stored at –80°C in RTL lysis buffer (RNeasy micro kit, Qiagen) with β-mercaptoethanol (1:100). For flow cytometry, Tregs were washed in PBS before staining. For further in vitro culturing, Tregs were collected in culture medium as described below.

### Suppression assay.

For all suppression assays, Teff were labeled with CellTrace Violet (5 μM, Thermo Fisher Scientific) prior to culturing alone (2 × 10^4^ cells / well; referred to as 1:0; in U-bottomed 96-well plates) or together with Tregs. Cells were stimulated with MACS Treg Suppressor Inspector Beads (Miltenyi Biotec) in culture medium (RMPI-1640 [Lonza] supplemented with 10% FBS [Thermo Fisher Scientific], 1% nonessential amino acids, 1% sodium pyruvate, 50 U/mL penicillin and 50 U/mL streptomycin [all from Invitrogen]) for 5 days at 37°C and 5% CO_2_. Ex vivo Tregs were immediately cocultured after isolation. In the rescue experiments, Treg fractions were first treated with rapamycin (2 μM in DMSO, Sigma-Aldrich) or DMSO alone (vehicle; 1/200, Sigma-Aldrich) for 4 hours in a U-bottom 96-well plates at a density of 6 × 10^5^ cells/100μL/well. Before addition to Teff or to the Boyden chamber migration assay, Tregs were washed in culture medium. Ratios at which cells were combined are specified in the appropriate figure legends (Figure 2, Figure 6, and Figure 7). Gating strategy is depicted in Supplemental Figure 3. Proliferation controls were included and consisted of Teff cultured alone at the highest cell density. As a quality control, threshold for donor inclusion was set at minimal 70% proliferation for the 1:0 condition. Next, using flow cytometry, cell viability and CellTrace Violet dilution were analyzed after 5 days. Prior to flow cytometry staining, culture supernatant was collected and stored at –80°C.

### Bulk RNA-Seq.

mRNA was isolated from Boyden chamber migration assay–derived Treg pellets from 5 HD and 4 persons with uRRMS using RNeasy micro kit (Qiagen) according to manufacturer instructions. RNA concentration and quality were determined using a NanoDrop spectrophotometer (Isogen Life Science). Per sample, an amount of 1 ng of total RNA was used as input for the SMART-Seq Stranded Kit (catalog 634444; protocol version 022829; low input option, Takara). This kit can deal with degraded as well as high-integrity input RNA. Positive and negative controls were included in the experimental design using 1 ng Control RNA (included in the kit) and 7 μL RNase inhibitor (RRI) water, respectively. First, RNA was converted to cDNA using random priming (scN6 Primer) and SMART (Switching Mechanism At 5’ end of RNA Template) technology, and then full-length adapters for Illumina sequencing (including specific barcodes for dual-indexing libraries) were added through PCR using a limited number of cycles (5). The PCR products were purified, and then ribosomal cDNA was selectively depleted by cleaving the ribosomal cDNAs by scZapR in the presence of mammalian-specific scR-Probes, which target nuclear and mitochondrial rRNA sequences. This process leaves the library fragments originating from non-rRNA molecules untouched. The remaining cDNA fragments were further amplified with primers universal to all libraries (14 cycles). Lastly, the PCR products were purified once more to yield the final cDNA library. All libraries were finally quantified using Qubit dsDNA HS kit (Thermo Fisher Scientific), and their size distribution was checked using a Bioanalyzer 2100 (Agilent). Sequence libraries of each sample were equimolarly pooled and sequenced on Illumina NovaSeq 6000 (100 bp, Single Reads, v1.5, 100-8-8-0) at the VIB Nucleomics Core (www.nucleomics.be). Quality of saw sequence reads was checked using FastQC version 0.11.8, and nucleotide calls with a quality score of 28 or higher were considered high quality. Adapters were removed using cutadapt v.2.4. Reads were aligned to the hg19 genome reference, using STAR (2.2.0e), and a maximum of 5 mismatches were allowed. Gene counts were retrieved using htseq-count using the “union” option. After removing absent features (zero counts in all samples), the raw counts were imported to R/Bioconductor package DESeq2 v.3.9 to identify DEGs among samples. The default DESeq2 options were used, including log fold change shrinkage. DEGs were considered only significant when the Benjamini-Hochberg adjusted *P* value (FDR) was < 0.05. Heatmaps were created using the gplots:heatmap.2() function on transformed raw counts.

IPA (Qiagen) was used to map lists of significant genes (FDR < 0.05) to gene ontology groups and biological pathways. The functional and canonical pathway analysis was used to identify the significant biological functions and pathways. Functions and pathways with *P* values less than 0.05 (Fischer’s exact test) were considered statistically significant.

The GSEA was done using GSEA software, which uses predefined gene sets from the Molecular Signatures Database (MsigDB). For the present study, we used all the H: hallmark gene sets for GSEA and a list of ranked genes based on a score calculated as log_10_ of *P* value multiplied by sign of fold change. The minimum and maximum criteria for selection of gene sets from the collection were 10 and 500 genes, respectively (74).

### Bead-based immunoassay.

hCMEC/D3 were trypsinized and cultured in collagen-coated 24 well plates (Greiner Bio-One) at a density of 25 × 10^3^ cells/cm^2^. After reaching near confluency (90%), cells were replenished with experimental medium as described before. One day later, cells were left untreated or were treated with TNF-α (100 ng/mL) and IFN-γ (10 ng/mL, both from Peprotech) for 24 hours in reduced medium as described before. After 24 hours, hCMEC/D3 cells were washed once and put on fresh reduced medium. After another 24 hours, supernatant were collected and stored at –80°C. To look at cytokine production by Tregs and Teff, the supernatants of suppression assays were collected after 5 days and stored at –80°C. Samples from BBB-ECs and cocultures were thawed and used undiluted in LEGENDplex Human Anti-Virus Response Panel (BioLegend) according to manufacturer instructions to measure different cytokines. Samples were acquired using the LSRFortessa (BD Biosciences) and analyzed using LEGENDplex Data Analysis Software (BioLegend). Concentrations were calculated by the use of the standard curves following the software guidelines.

### qPCR.

To determine gene expression levels, hCMEC/D3 were trypsinized and cultured in 24-well plates (Greiner Bio-One) at a density of 25 × 10^3^ cells/cm^2^. After reaching near confluency (90%), cells were replenished with experimental medium as described before. The day afterward, cells were left untreated or were treated with TNF-α (100 ng/mL) and IFN-γ (10 ng/mL, all Peprotech) for 24 hours in reduced medium as described before. After 24 hours, hCMEC/D3 cells were washed once and put on fresh reduced medium. After another 24 hours, cell pellets were collected and stored at –80°C. Cells were lysed using RLT lysis buffer (Qiagen) with β-mercaptoethanol (1:100), and RNA was extracted using RNeasy micro kit (Qiagen), according to the manufacturer instructions. mRNA concentration was measured with Nanodrop (Thermo Fisher Scientific). Conversion to cDNA was performed using qScript cDNA supermix (Quanta Biosciences). Quantitative PCR (qPCR) was performed on a StepOne detection system (Thermo Fisher Scientific). Primer sequences are listed in the supplementary information (Supplemental Table 4). Relative quantification of gene expression was accomplished using the comparative Ct method. Data were normalized to the most stable reference genes using geNorm.

### Flow cytometry.

PBMCs were obtained via ficoll density gradient centrifugation (Cederlane lympholyte) for 20 minutes at 800*g* and room temperature without brake and were frozen in liquid nitrogen for storage (10% DMSO in FBS; Thermo Fisher Scientific). At the time of analysis, samples were thawed and rested for 4 hours at 37°C, 5% CO_2_ culture medium. Twenty-six HD, 24 people with uRRMS, 25 people with flRRMS, and 23 people with uSPMS were included. Fresh paired CSF and blood samples of 5 people with uRRMS were used immediately for phenotyping. Treg fractions collected from the Boyden chamber assay were used directly for staining. For cytokine expression, cells were stimulated with PMA (Merck), ionomycin (Merck), and Golgiplug (BD Biosciences) for 4 hours at 37°C at 5% CO_2_. After live/dead staining using Fixable Viability Dye eFluor 506 or LIVE/DEAD Fixable Aqua Dead Cell Stain Kit (Thermo Fisher Scientific), the following antibodies were used: CD25 BB515 (2A3; BD Biosciences); CD127 PerCP/Cy5.5 (A019D5), Helios PerCP/Cy5.5 (22F6), FOXP3 BV421 (206D), CCR5 PE (3A9), CD11a AF594 (HI111), CCR6 BV650 (G034E3), CCR6 PE/Cy7 (G034E3), CD49d BV605 (9F10), CXCR3 BV711 (G025H7), MCAM APC (P1H12), CD3 AF700 (HIT3a), IL-6R PE/Cy7 (UV4), CCR4 PE/Dazzle 594 (L291H4), IFN-γ PerCP/Cy5.5 (4S.B3), and TNF-α PE/Cy7 (all from BioLegend); and AREG APC (AREG559) and CD4 APC-eFluor780 (OKT4; both from Thermo Fisher Scientific). Cells were permeabilized using the FOXP3/Transcription factor staining buffer kit (Thermo Fisher Scientific) according to manufacturer instructions. Human BD Fc block (BD Biosciences) was also used for paired blood-CSF staining. Samples were acquired using the LSRFortessa (BD Biosciences) and Aurora (Cytek) and were analyzed using FlowJo 10.8.0 (BD Biosciences) and OMIQ (FlowSOM analysis, R path for FlowSOM visualization; Supplemental Material). Percentages and median fluorescence intensity (MFI) are given.

### mRNA transfection.

Human Tregs were sorted by FACS from HD-derived PBMCs and cultured for 6 days in the presence of IL-2 (150 ng/mL; Peprotech), anti-CD3 (10 μg/mL; OKT3, Invitrogen), and anti-CD28 (1 μg/mL; CD28.2, BD Biosciences). Next, cells are washed and left to rest for 24 hours with IL-2 (50 ng/mL). Then, *AREG* mRNA (1.25 μg/1 × 10^6^ cells, provided by eTheRNA) or water (mock) was transfected into the cells using electroporation (500V, 5 ms, single square wave pulse) with the ECM 830 electroporation system (BTX) and followed for 4 days. Percentage of AREG^+^ Tregs by flow cytometry and AREG production in supernatant using an ELISA were checked daily.

### AREG ELISA.

Blood was collected in heparin-coated tubes and centrifuged for 10 minutes at 1,500*g* and room temperature to collect plasma. CSF samples were collected and centrifuged at 500*g* and 4°C for 10 minutes to select CSF liquor. Both were stored at –80°C. *AREG* mRNA-transfected Tregs and paired plasma-CSF samples of people with uRRMS were analyzed using the Human Amphiregulin Quantikine ELISA Kit (R&D systems) according to the manufacturer instructions. Absorbance was measured at 450 and 570 nm using a CLARIOstar plus (BMG Labtech).

### Statistics.

Statistical analyses were performed using GraphPad Prism version 10.1.2 (GraphPad Software). Details of statistical tests are given in figure legends. Tests used included: 2-way ANOVA with Bonferroni’s multiple-comparison test, (1-tailed) Wilcoxon test, Friedman test, Kruskal-Wallis with Dunn’s multiple-comparison test, and the Mann-Whitney *U* test. Cumulative data are shown as mean ± SEM. *P* < 0.05 was considered significant.

### Study approval.

All mouse procedures were in accordance with the EU directive 2010/63/EU and were approved by the Hasselt University Ethics Committee for Animal Experiments (local identification nos. 201811, 201812, 202045, and 202309). Donating participants signed informed consent, and study approval was obtained by the local ethical committees (Hasselt University; local identification CME2016/687).

### Data availability.

Next-generation sequencing data have been uploaded to GEO under the accession nos. GSE255171 (bulk RNA-Seq) and GSE255607 (scRNA-Seq). Values for all data points in graphs are reported in the Supporting Data Values file.

## Author contributions

Conceptualization was contributed by BB; methodology was contributed by PB, IH, CH, LA, and MH; formal analysis was contributed by PB, IH, LA, and MH; investigation was contributed by PB, LA, and MH; recruitment of participants was contributed by BVW, VP, VS and TK; writing of the original draft was contributed by PB; review and editing of the manuscript were contributed by BB, NH, TK, and MK; visualization was contributed by PB and IH; and supervision was contributed by MK, BB, and NH. All authors have read and agreed to the published version of the manuscript.

## Supplementary Material

Supplemental data

Supplemental data set 1

Supporting data values

## Version 1 02/22/2024

In-Press Preview

## Version 2 04/08/2024

Electronic publication
