# Supplementary material for: Rapamycin rescues loss of function in blood-brain barrier–interacting Tregs
Source: JCI Insight. 2024 Feb 22;9(7):e167457. doi: 10.1172/jci.insight.167457 (PMC11128200; doi:10.1172/jci.insight.167457)
Supplement: Supplemental data [file jciinsight-9-167457-s211.pdf]

Rapamycin rescues loss-of-function in blood-brain barrier-interacting regulatory T cells

Paulien Baeten[1,2], Ibrahim Hamad[1,3], Cindy Hoeks[1,2], Michael Hiltensperger[4], Bart Van Wijmeersch[5,6], Veronica Popescu[5,6], Lilian Aly[4], Veerle Somers[1,2], Thomas Korn[4,7], Markus Kleinewietfeld[1,3], Niels Hellings[1,2,#], Bieke Broux[1,2,#,*]

Supplementary Figures and Tables

Supplementary Figures 1-13

Supplementary Tables 1-4

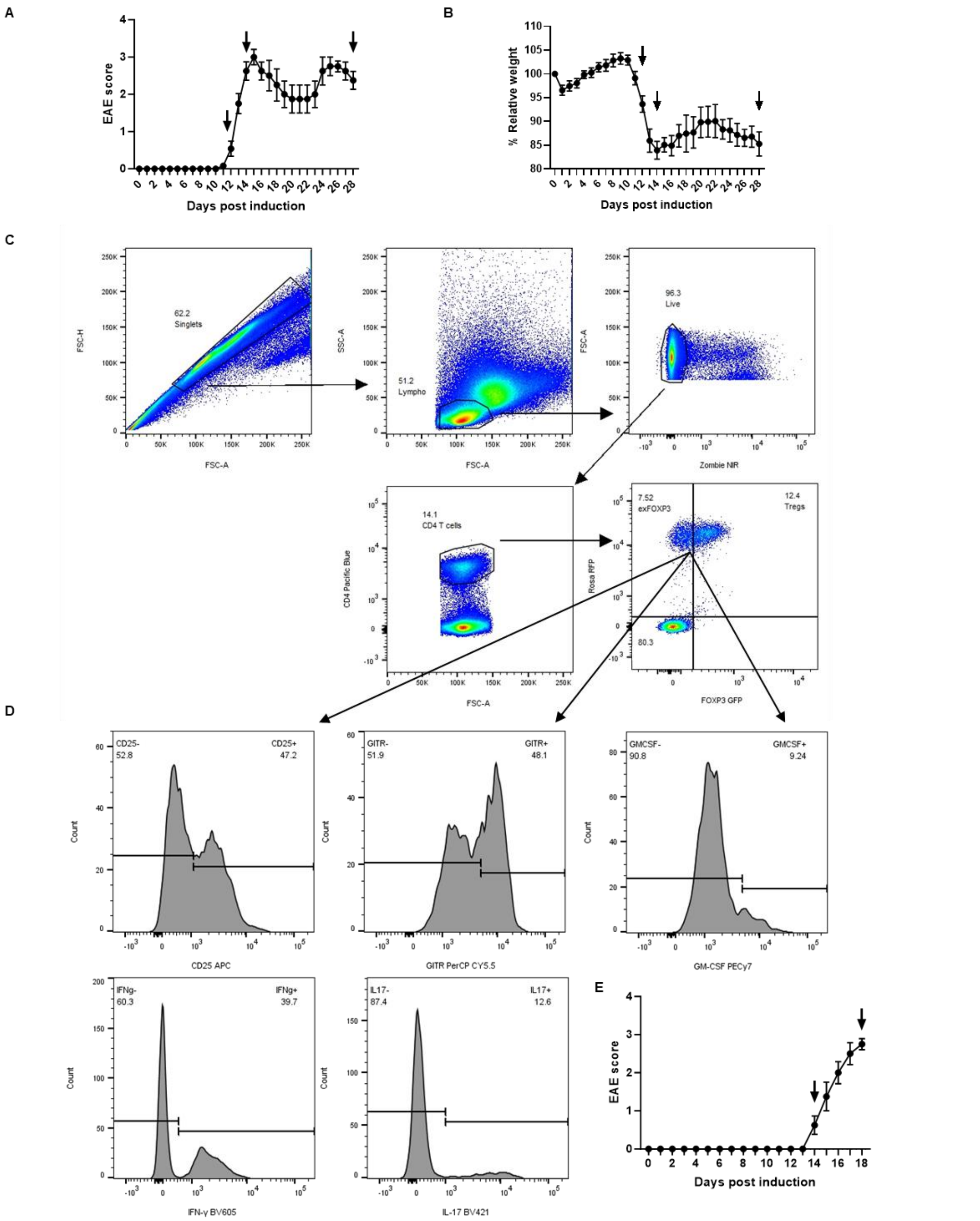

Fig. S1. Disease development and gating strategy of FOXP3$^+$ Tregs and exFOXP3 T cells *in vivo*. A-B. Course (A) of EAE with onset (12 dpi, arrow), peak (14 dpi, arrow) and chronic (28 dpi, arrow) correlates to weight loss (B). Relative weight is normalized to weight on day of induction (100%). n=4-12. **C**. Isolated cells were used for flow cytometry. Using FOXP3$^{Cre\text{-}GFP}$ Rosa^{RFP} fate-mapping mice, RFP$^+$GFP$^+$ FOXP3$^+$ Tregs and RFP$^+$GFP$^-$ exFOXP3 T cells were studied in EAE. Gating strategy and representative plots of FOXP3$^+$ Tregs and exFOXP3 T cells isolated from splenocytes are shown. **D**. Cells were stimulated for 4h with PMA, ionomycin and Golgiplug. RFP$^+$GFP$^+$ FOXP3$^+$ Tregs and RFP$^+$GFP$^-$ exFOXP3 T cells are gated as in panel C. Representative histograms of CD25$^+$, GITR$^+$, GM-CSF$^+$, IFN-γ$^+$ and IL-17$^+$ within exFOXP3 T cells in CNS at the chronic phase of the disease. **E**. Course of EAE with onset (14 dpi, arrow) and peak (18 dpi, arrow). Isolated cells were used for single-cell RNA-seq. RFP$^+$GFP$^+$ FOXP3$^+$ Tregs and RFP$^+$GFP$^-$ exFOXP3 T cells were FACS-sorted for single-cell RNA-seq as shown in panel C. n=4

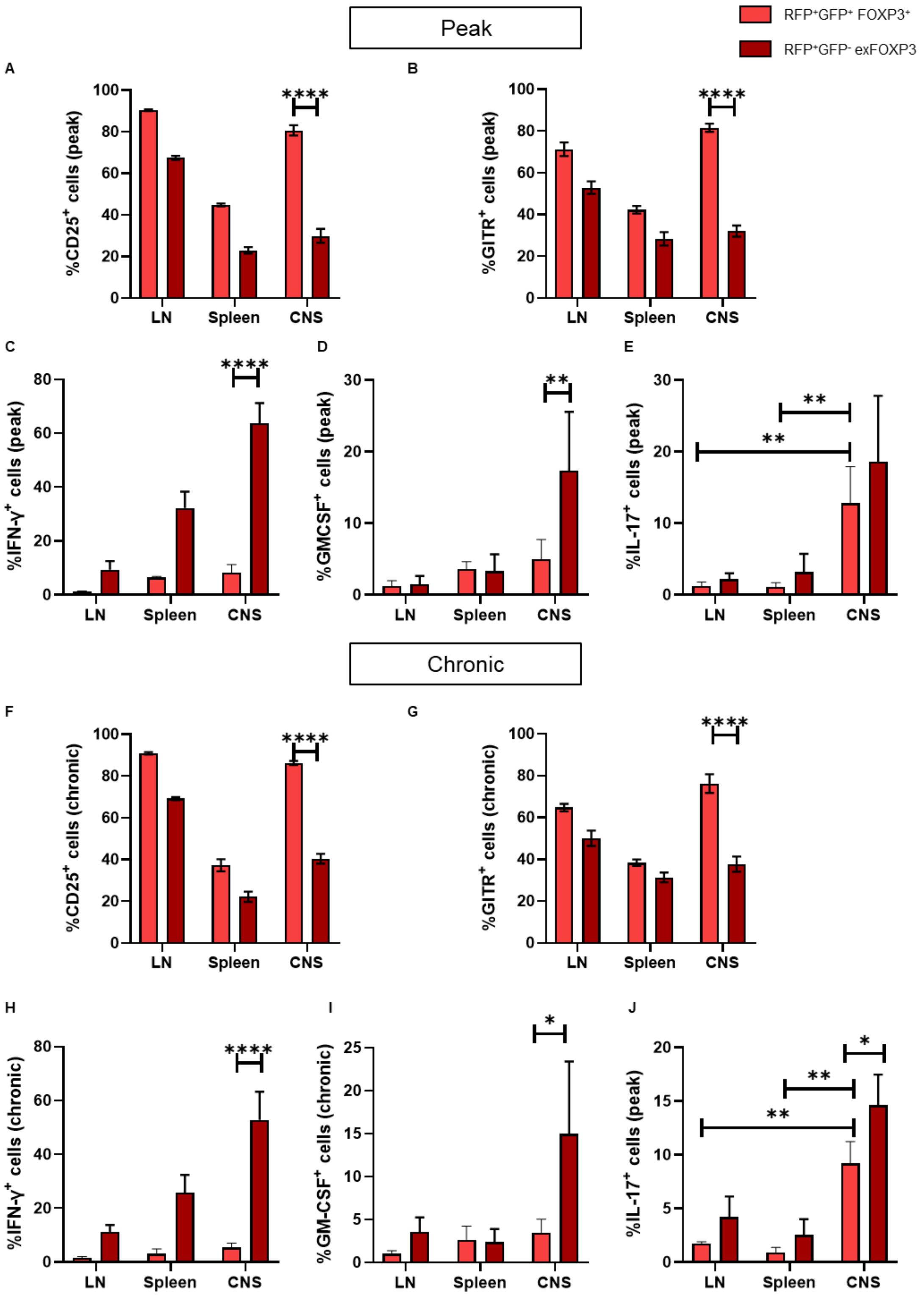

Fig. S2. Frequency of Treg markers and inflammatory cytokines in FOXP3$^+$ Tregs and exFOXP3 T cells in periphery and CNS. EAE was induced in female FOXP3$^{Cre\text{-}GFP}$ Rosa^{RFP} fate-mapping mice. Tissues were collected at peak or chronic phase (Fig. S1) of disease and immune cells were isolated from spleen, lymph nodes (LN) or pooled brain and spinal cord (CNS) for flow cytometry. Isolated cells were stimulated for 4h with PMA, ionomycin and Golgiplug. Percentage of CD25$^+$ (A+F), GITR$^+$ (B+G), IFN-γ$^+$ (C+H), GM-CSF$^+$ (D+I) and IL-17$^+$ (E+J) within either FOXP3$^+$ Tregs or exFOXP3 T cells in the different tissues at different stages of the disease. Representative dot plot of RFP vs. GFP in splenocytes and gating strategy in Fig. S1. n=4; 2way ANOVA with Bonferroni's multiple comparisons test. Data are plotted as mean ± SEM. *: $p<0.05$; **: $p<0.01$; ***: $p<0.001$; ****: $p<0.0001$.

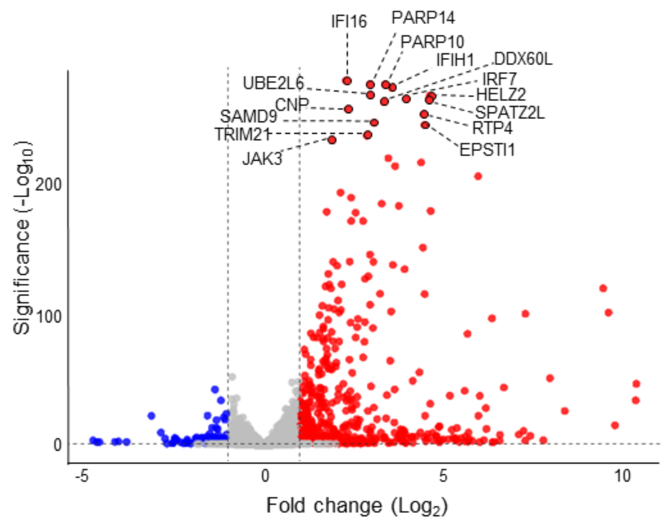

Fig. S3. JAK3/STAT pathway related genes are upregulated in Tregs that interacted with BBB-ECs compared to untouched Tregs. HD-derived Tregs were loaded on an inflamed Boyden chamber migration assay as represented in Fig. 2A. Tregs were also cultured alone for 24h in the same EC medium (untouched Tregs). After 24h, untouched, migrated and nonmigrated Tregs were collected and bulk RNA-seq was performed. To identify the changes induced by contact with BBB-ECs, the genes that were shared (not significantly different) between migrated and nonmigrated Tregs were compared with those in untouched Tregs. X-axis shows the log2 fold change for ratio shared migrated and nonmigrated/untouched. Y-axis shows statistical significance (FDR-adjusted p-value). Up- and downregulated genes are coloured red or blue respectively when adjusted p-value<0.05 and |log2FoldChange|>1. Top DEGs are highlighted. n=5 (HD)

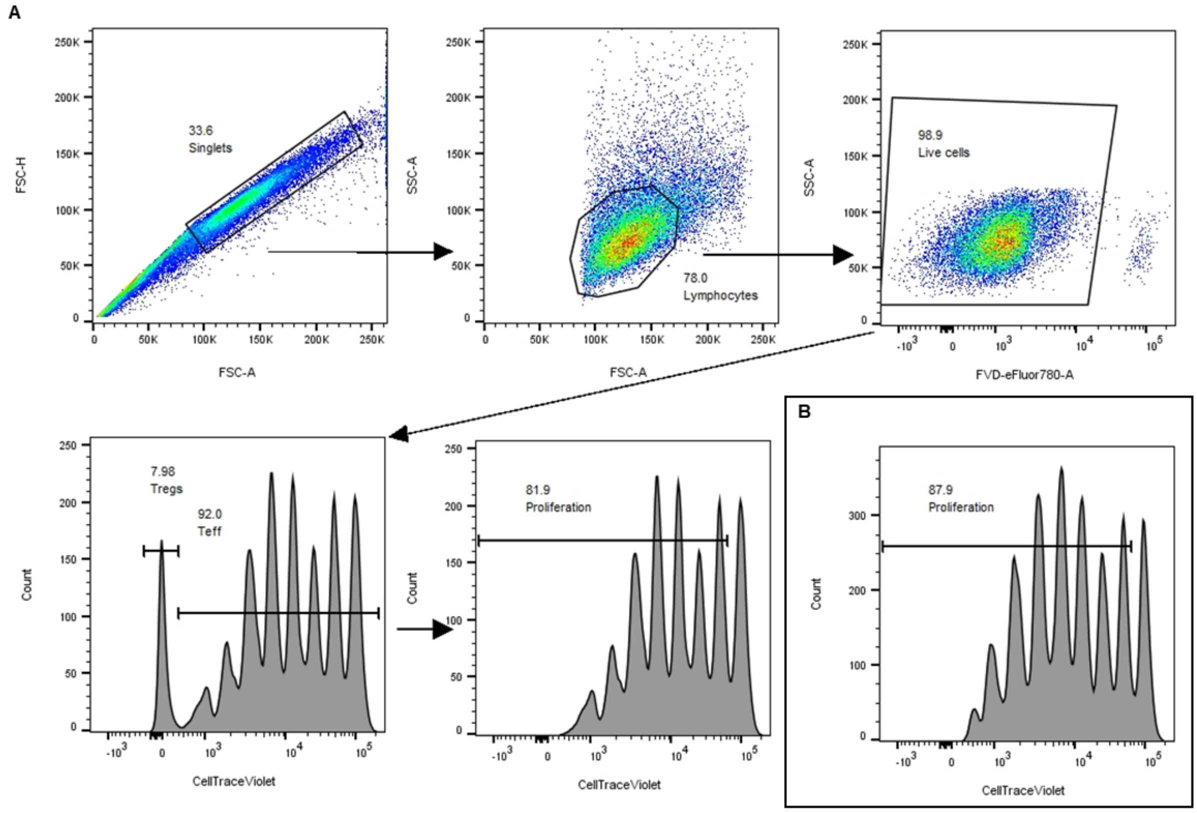

Fig. S4. Gating strategy of suppression assay. Stimulated CellTrace Violet-labelled Teff are cultured for 5 days with and without Tregs. The dilution of the CellTrace is used as a measurement for proliferation. Gating strategy and representative plots of Teff proliferation cultured with migrated Tregs (A, 1:1) or cultured alone (B, 1:0).

Table S1: Pathway analysis of migrated vs. nonmigrated Tregs using ingenuity pathway analysis (IPA).

Top 10 IPA pathways	HD			uRRMS		
	Rank	-log (p-value)	z-score	Rank	-log (p-value)	z-score
EIF2 Signaling	1	37.5	-6.274	1	41.9	-6.14
mTOR Signaling	2	15.28	-0.928	3	17.9	-0.333
Regulation of eIF4 and p70S6K Signaling	3	15.25	-1.606	4	16.4	-0.853
Coronavirus Pathogenesis Pathway	4	14.9	4.695	2	18.9	4.529
Th2 Pathway	5	10.1	0.87	6	10.3	1.667
Th1 and Th2 Activation Pathway	6	9.67	ND	5	10.7	ND
Molecular Mechanisms of Cancer	7	8.81	ND	8	9.29	ND
Senescence Pathway	8	7.24	1.912	7	9.37	1.789
RHOA Signaling	9	7.17	2.466		4.74	2.401
Protein Kinase A Signaling	10	7.04	-2.39		4.49	-2.151
ILK Signaling	11	7	-0.729		5.97	0.745
PD-1, PD-L1 cancer immunotherapy pathway	12	6.79	-2.694	10	8.29	-2.744
IL-4 Signaling	13	6.23	ND		4.73	ND
Hepatic Fibrosis / Hepatic Stellate Cell Activation	14	6.21	ND		6.2	ND
Epithelial Adherens Junction Signaling	15	5.82	1.508	14	7.68	1.697
Th1 Pathway	16	5.77	2.646	20	6.56	3.182
Axonal Guidance Signaling	17	5.68	ND	13	7.84	ND
Actin Cytoskeleton Signaling	18	5.62	2.714	18	6.83	3.434
Pulmonary Fibrosis Idiopathic Signaling Pathway	19	5.58	1.287	12	8.03	2.4
ID1 Signaling Pathway	20	5.56	0.555		ND	ND
Role of Tissue Factor in Cancer		4.64	0.287	9	8.33	ND
Role of Macrophages, Fibroblasts and Endothelial Cells in Rheumatoid Arthritis		3.72	0.212	11	8.24	ND
Telomerase Signaling		ND	ND	15	7.07	0
Hepatic Fibrosis Signaling Pathway		ND	ND	16	6.92	2.132
Chronic Myeloid Leukemia Signaling		3.37	0.264	17	6.83	ND
p70S6K Signaling		4.71	0.279	19	6.69	-1.121
Cell Cycle: G1/S Checkpoint Regulation		3.6	0.289	20	6.59	-1.091

ND: not determined

Table S2: Pathway analysis of migrated vs. nonmigrated Tregs using gene set enrichment analysis (GSEA).

**Top 20 GSEA Hallmark pathways**	**HD**			**uRRMS**		
	**Rank**	**NES**	**FDR**	**Rank**	**NES**	**FDR**
Hallmark_Epithelial_Mesenchymal_Transition	1	2.852776	0.0001	1	2.6852849	0.0001
Hallmark_Inflammatory_Response	2	2.1911776	0.0001	13	1.9546956	0.0001
Hallmark_MYC_Targets	3	-2.170037	0.0001	9	-2.0970435	0.0001
Hallmark_G2M_Checkpoint	4	2.1378007	0.0001	2	2.2614634	0.0001
Hallmark_Mitotic_Spindle	5	2.1286461	0.0001	6	2.1852949	0.0001
Hallmark_Complement	6	2.1181417	0.0001	11	2.0063581	0.0001
Hallmark_Cholesterol_Homeostasis	7	2.0137348	0.0001	8	2.171311	0.0001
Hallmark_P53_Pathway	8	2.0074844	0.0001	12	1.9742134	0.0001
Hallmark_Myogenesis	9	2.005464	0.0001		*1.59*	*0.007*
Hallmark_TNFA_Signaling_via_NFKB	10	1.9876082	0.0001	7	2.1851656	0.0001
Hallmark_Apical_Junction	11	1.9812022	0.0001	20	1.8020948	0.001
Hallmark_E2F_Targets	12	1.9573711	0.0001		*1.73*	*0.002*
Hallmark_Reactive_Oxygen_Species_Pathway	13	1.9182377	0.0001	3	2.2454598	0.0001
Hallmark_Hypoxia	14	1.9018121	0.0001	10	2.060272	0.0001
Hallmark_mTORC1_Signaling	15	1.8804277	0.001	5	2.2114327	0.0001
Hallmark_Apoptosis	16	1.868929	0.0001	4	2.2446556	0.0001
Hallmark_Coagulation	17	1.8672571	0.0001		*1.36*	*0.039*
Hallmark_Allograft_Rejection	18	1.84033	0.001	15	1.9102324	0.0001
Hallmark_Interferon_Gamma_Signaling	19	1.8319241	0.001		*1.75*	*0.002*
Hallmark_Kras_Signaling	20	1.8258758	0.001		*1.50*	*0.013*
Hallmark_Angiogenesis		*1.59*	*0.008*	14	1.9246495	0.0001
Hallmark_UV_Response		*1.8*	0.001	16	1.8945185	0.0001
Hallmark_IL6_JAK_STAT3_Signaling		*1.55*	*0.01*	17	1.8220509	0.001
Hallmark_IL2_STAT5_Signaling		*1.7*	*0.003*	18	1.8084469	0.001
Hallmark_PI3K_Akt_mTOR_Signaling		*1.37*	*0.047*	19	1.8031695	0.001

NES: normalized enrichment score

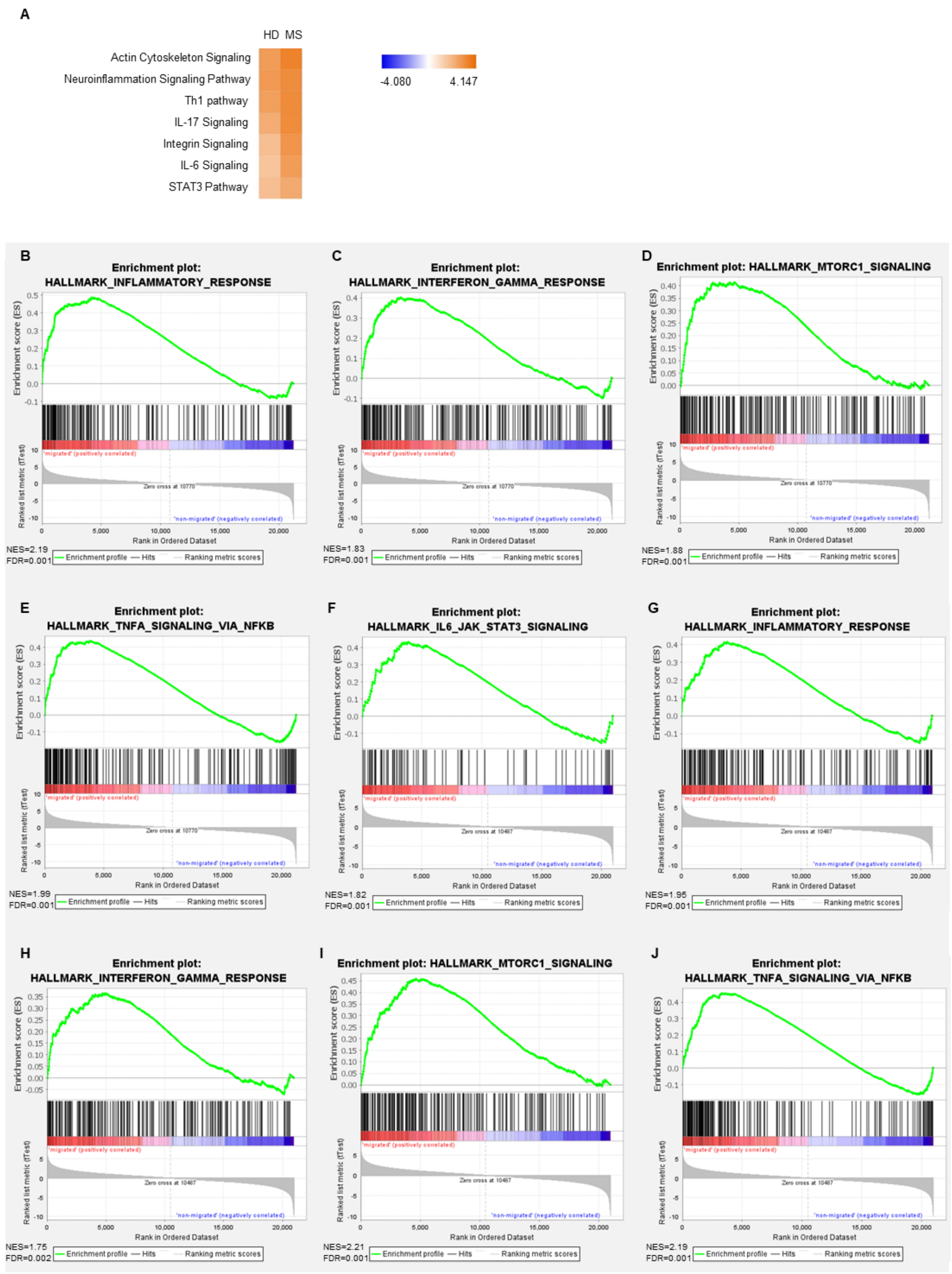

Fig. S5: Pathway analysis using IPA and GSEA of transcriptomes of migrated vs. nonmigrated Tregs isolated from HD and people with uRRMS. A. IPA predictions of canonical pathways on comparison analysis of migrated vs. nonmigrated Tregs. Z-score predicts whether a canonical pathway is increased (positive z-score, orange) or decreased (negative, blue) in accordance with experimental datasets. Fischer's exact test. **B-J.** GSEA enrichment plots (KEGG pathways) of inflammatory response, IFN-γ response, mTORC1 signaling, TNF-α signaling and IL-6-STAT3 signaling of migrated and nonmigrated Tregs isolated from HD (**B-E**) and people with uRRMS (**F-J**). The green curve corresponds to the ES curve, which represents the running sum of the weighted enrichment score obtained from the GSEA algorithm. The red-blue band represents the degree of correlation of genes with the depicted pathway (red for positive and blue for negative correlation). The NES and corresponding FDR are reported within the plot. ES: enrichment score; KEGG: Kyoto encyclopedia of genes and genomes

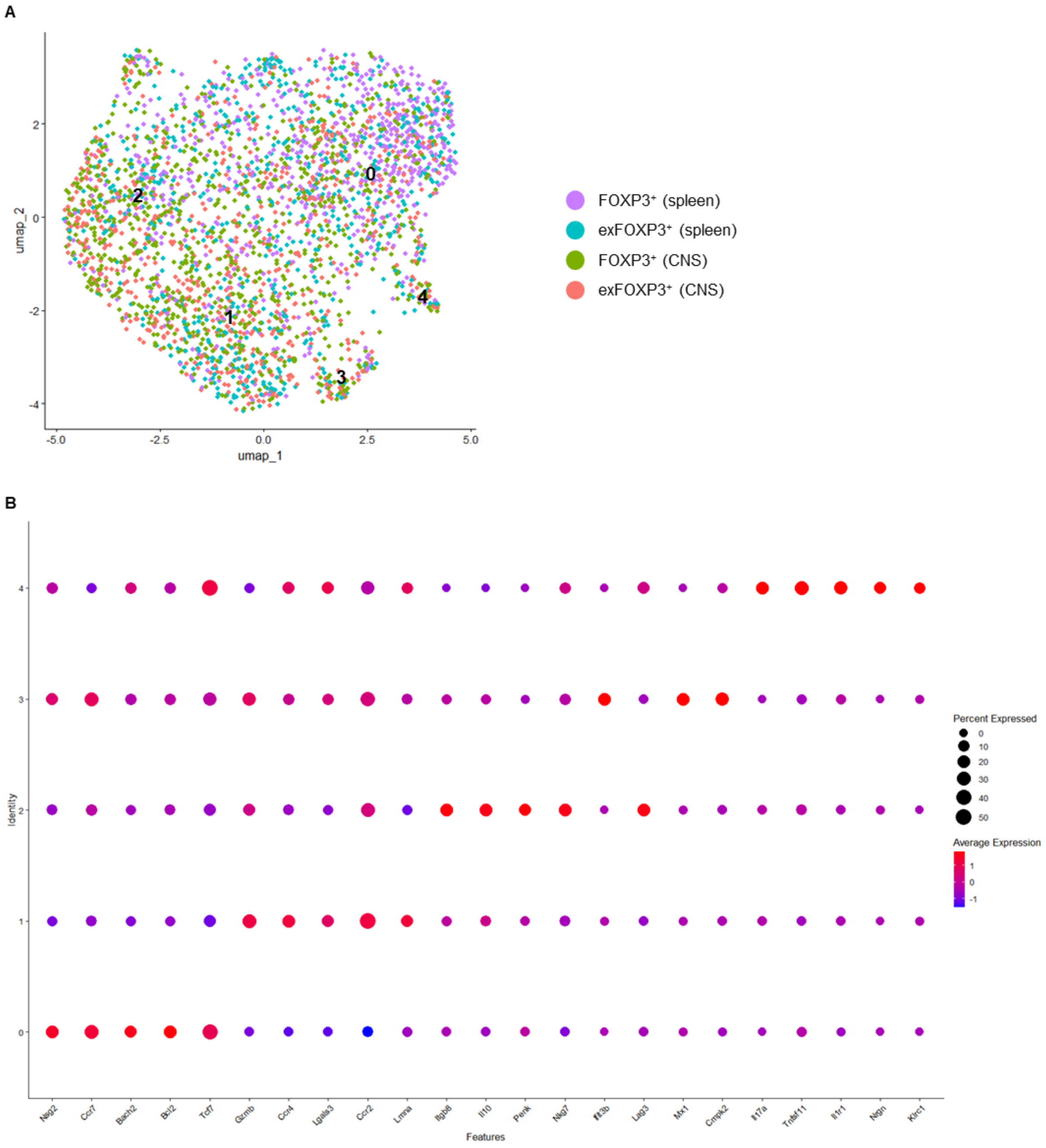

Fig. S6. Inflammatory genes are upregulated in CNS-isolated Tregs. EAE was induced in female FOXP3$^{Cre\text{-}GFP}$ Rosa^{RFP} fate-mapping mice. Tissues were collected at peak (18 dpi, Fig. S1) and RFP^{+}GFP^{+} FOXP3^{+} Tregs and RFP^{+}GFP^{-} exFOXP3 T cells were FACS-sorted from spleen and pooled brain and spinal cord (CNS). Gating strategy in Fig. S1. Single-cell RNA-seq was performed on CNS- and spleen-derived RFP^{+}GFP^{-} exFOXP3 T cells and RFP^{+}GFP^{+}FOXP3^{+} Tregs. **A**. Two-dimensional UMAP plot showing the clustering of 3029 cells based on gene expression divided into 5 clusters. Point coordinates are based on UMAP dimensionality reduction of the top 15 principal components. Individual points correspond to single cells colored according to samples. **B**. Top expressing genes per cluster.

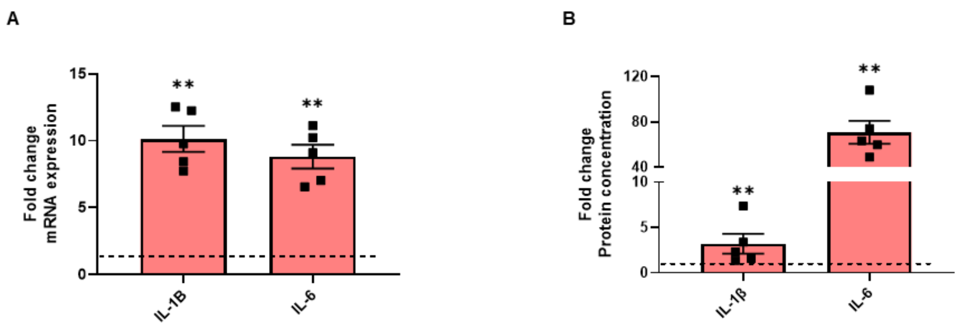

Fig. S7. hCMEC/D3 cells produce IL-6 and IL-1β. hCMEC/D3 cells were left untreated or were TNF-α (100 ng/ml) and IFN-γ (10 ng/ml) treated for 24h. After washing the inserts and replenishing with fresh medium, cell pellets and supernatant were collected after another 24h, representing the timeslot of Treg coculture. **A.** Fold change of *IL1B* and *IL6* mRNA expression shown compared to untreated cells (dotted line). n=5; Mann-Whitney test. **B.** Fold change of IL-1β and IL-6 concentration shown compared to untreated cells (dotted line). n=5; Mann-Whitney test. Data are represented as mean ± SEM. **: p<0.01.

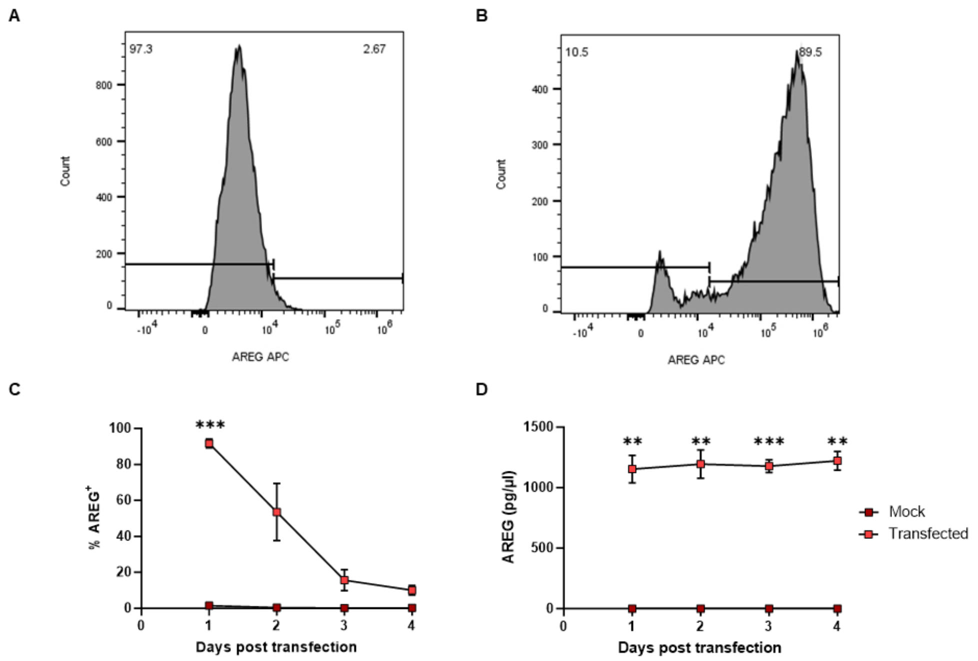

Fig. S8. mRNA-induced AREG overexpression in primary human Tregs. Human Tregs are FACS-sorted from HD-derived PBMCs and cultured for 6 days in the presence of IL-2, anti-CD3 and anti-CD28. Next, cells are washed and left to rest for 24h with IL-2. Then, *AREG* mRNA or water (mock) was transfected into the cells using electroporation and followed for 4 days. **A-C.** Using flow cytometry, the percentage of $AREG^+$ Tregs is determined. Showing $AREG^+$ Tregs at 1 day after mock (A) or AREG mRNA transfection (B) and during 4 days (C). **D.** Using ELISA, the production of AREG into the supernatant was determined. n=4; 2way ANOVA with Bonferroni's multiple comparisons test. **: p<0.01; ***: p<0.001.

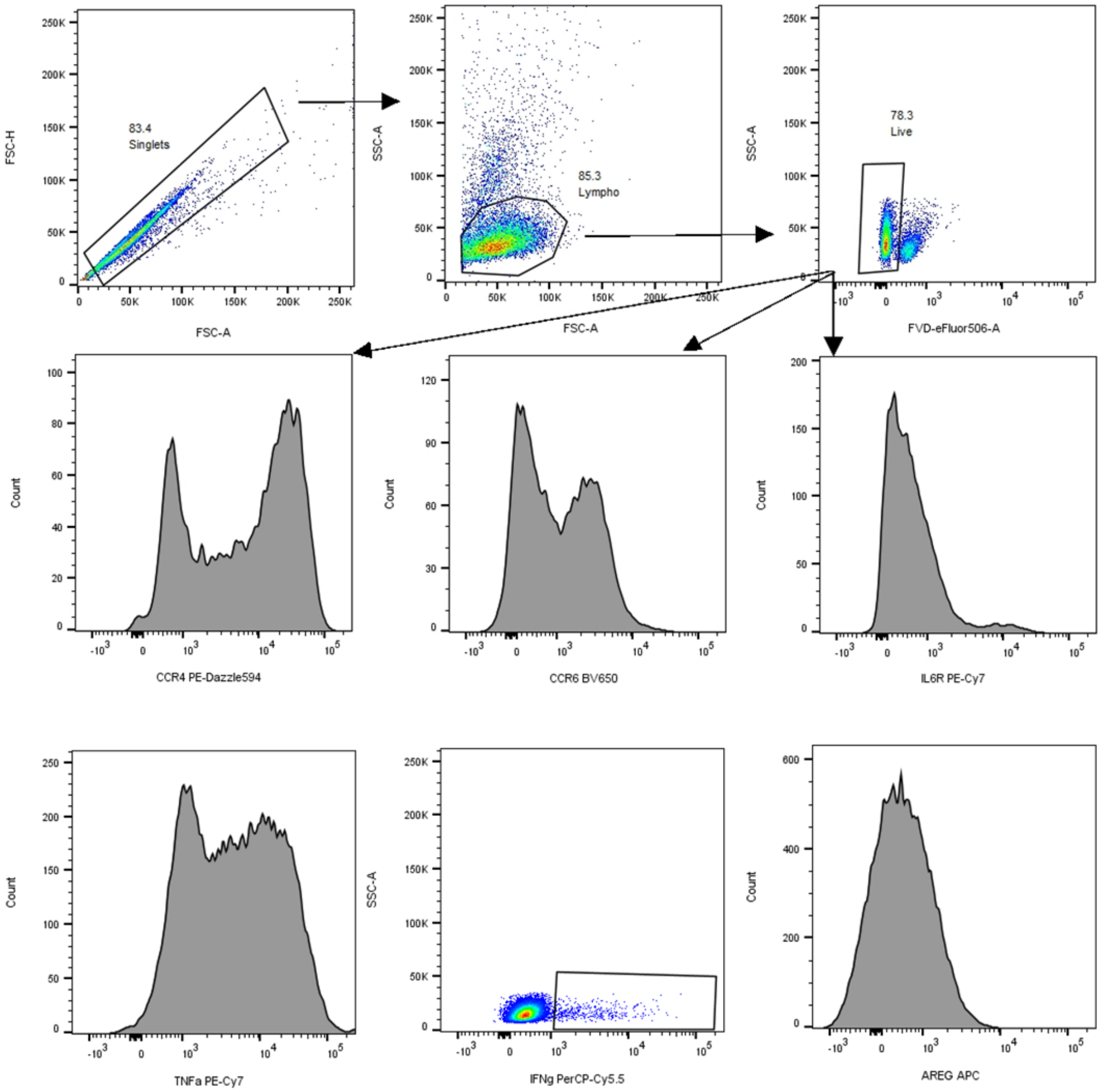

Fig. S9. Gating strategy of RNA-seq-identified hits on nonmigrated and migrated Tregs using flow cytometry. Tregs were loaded on a Boyden chamber migration and the 2 cell fractions were collected and stained. Gating strategy and representative plots of nonmigrated Tregs are shown.

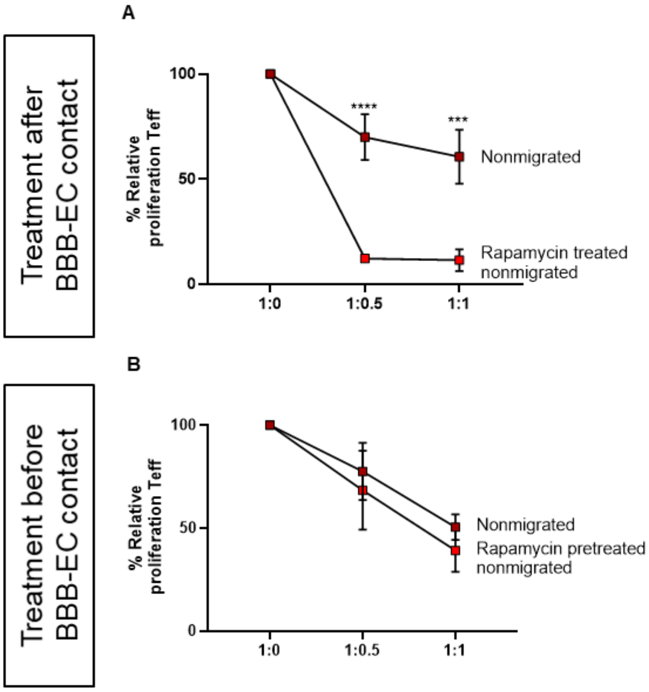

Fig. S10. Rapamycin boosts suppressive capacity of nonmigrated Tregs after interaction with inflamed BBB-ECs but is unable to maintain Treg function when treated before BBB-EC interaction. A. Nonmigrated HD-derived Tregs were collected and treated with rapamycin (2µM) for 4h, washed and cocultured with Teff in a suppression assay. n=5. **B.** HD-derived Tregs were treated with rapamycin (2µM) for 4h, washed and loaded on the Boyden chamber migration assay. After 24h, nonmigratedcells were collected and cocultured with Teff in a suppression assay. n=3. Percentage proliferation represents CellTrace dilution of Teff in suppression assays with different ratio's of Teff and Tregs (given as Teff:Tregs). Relative proliferation is normalized to 1:0 condition (100%). Gating in Fig. S4.; 2way ANOVA with Bonferroni's multiple comparisons test. Data are represented as mean ± SEM. ***: p<0.001; ****: p<0.0001.

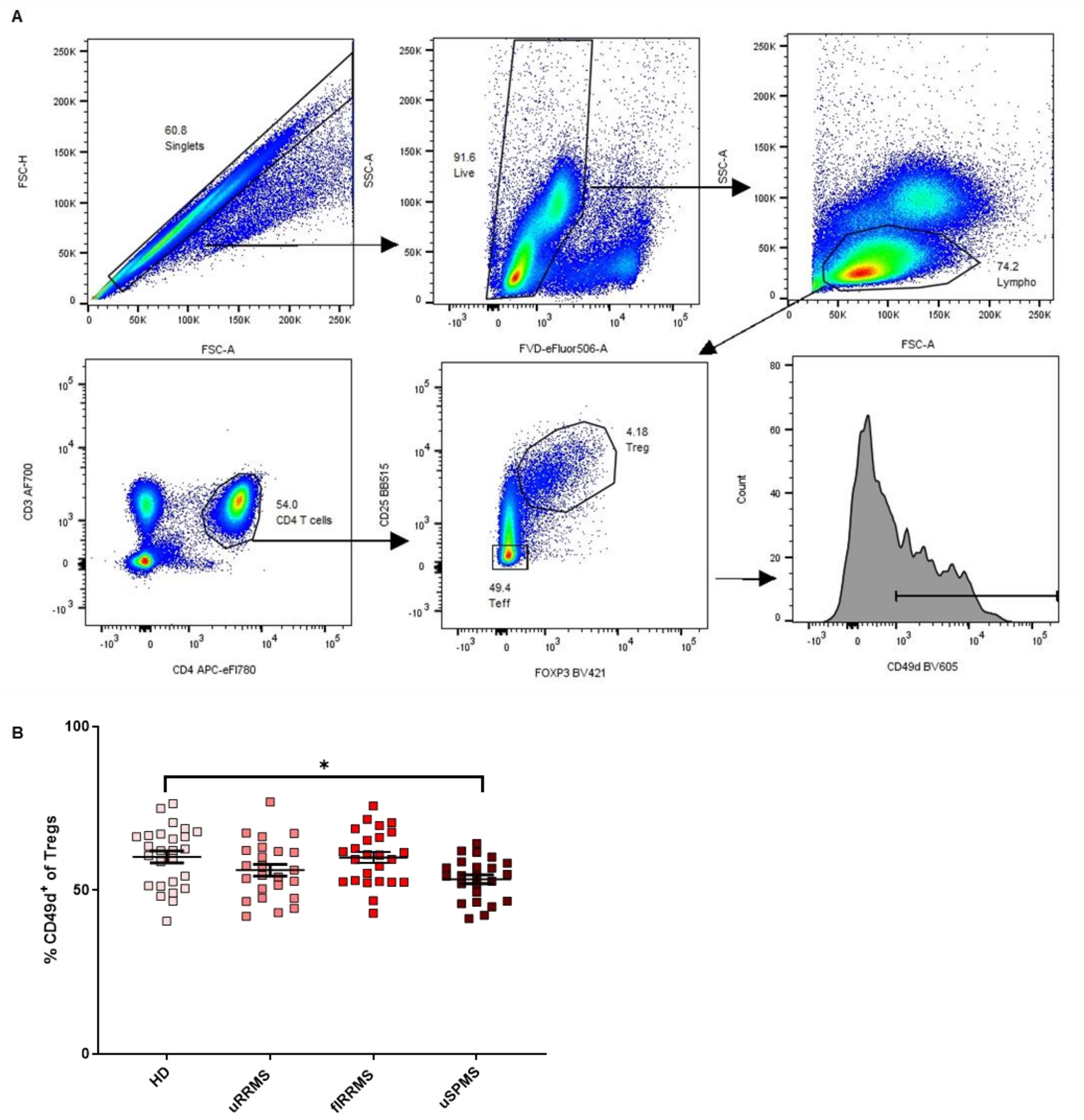

Fig. S11. Blood-derived Tregs do not show differences in CD49d expression by HD and people with MS. Frozen PBMCs of HD or people with uRRMS , flRRMS or uSPMS were thawed and studied with flow cytometry. **A.** Gating strategy and representative plots of Tregs are shown. **B.** % CD49d$^+$ Treg are not increased in MS types. Data are represented as mean ± SEM. n=26 (HD), n=24 (uRRMS), n=25 (flRRMS), n=23 (uSPMS); Kruskal-Wallis test with Dunn's multiple comparisons test, *: $p<0.05$.

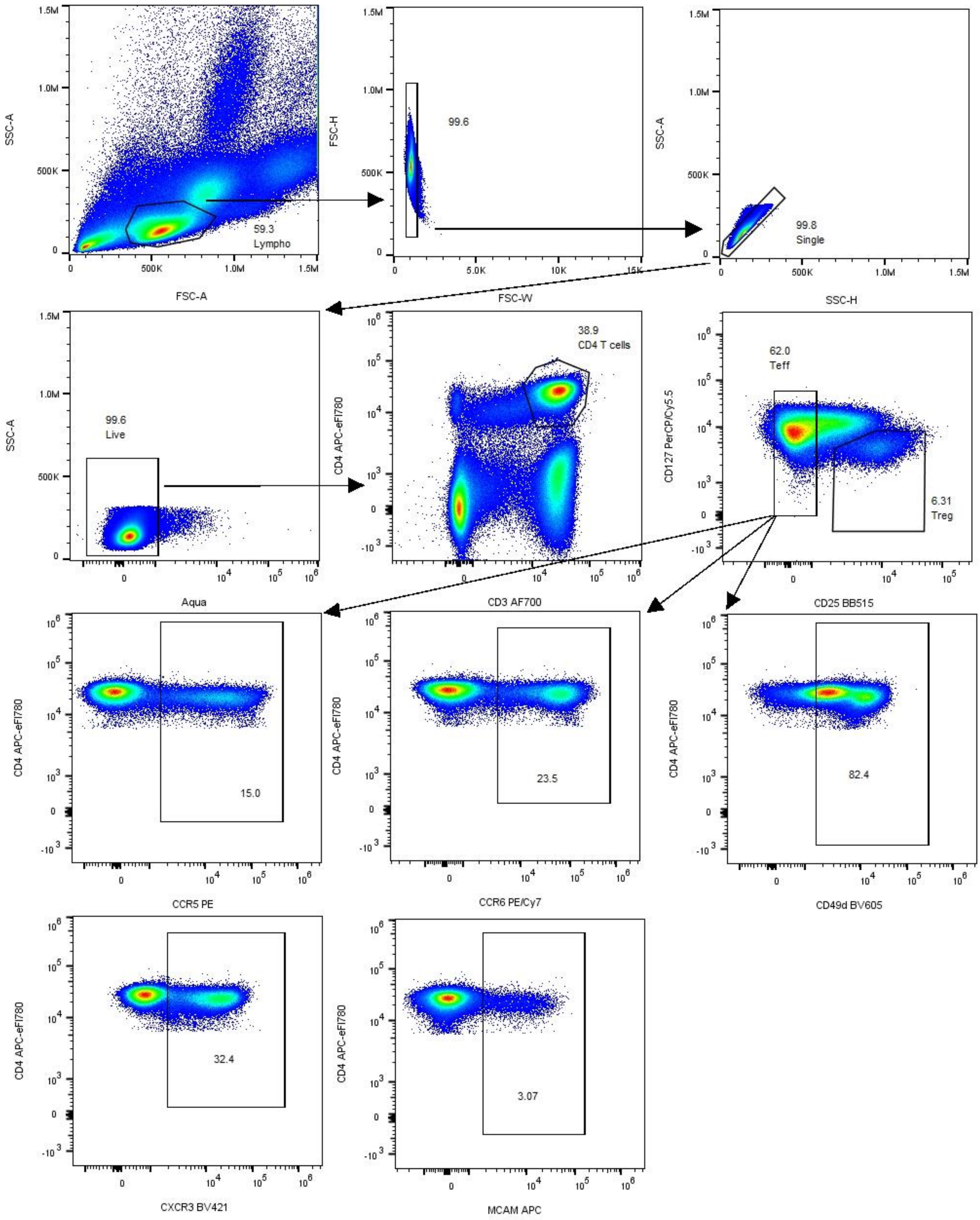

Fig. S12. Gating strategy of migratory molecules on Tregs and Teff from paired blood and CSF samples of people with uRRMS. Fresh PBMCs and CSF samples were taken to diagnose MS and studied with flow cytometry. Gating strategy and representative plots of peripheral blood Teff are shown of people with MS.

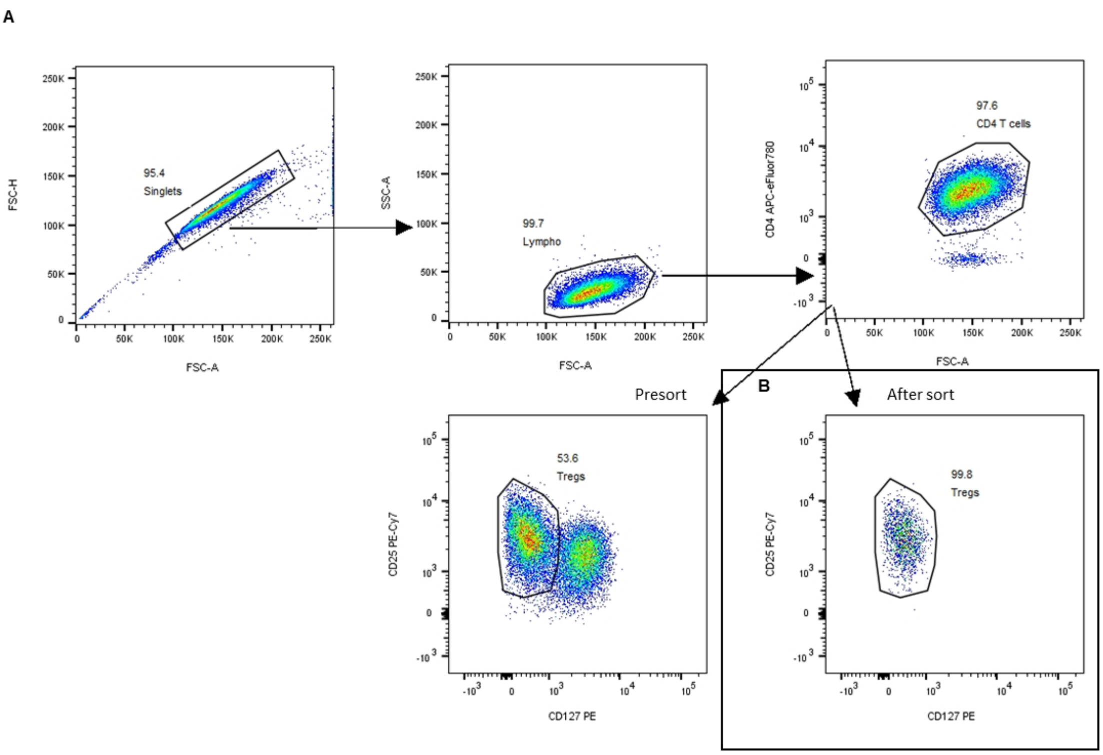

Fig. S13. Gating strategy of human Treg sorting. A. Fresh PBMCs were sorted for Tregs based on CD4, CD25 and CD127 expression. **B.** Purity of sorted $CD4^{+}CD25^{high}CD127^{low}$ Tregs.

Table S3: Donor information.

	**HD**	**Untreated RRMS**	**First-line treated RRMS**	**Untreated SPMS**
**Bulk RNA-seq**				
Number	5	4		
Gender (M/F)	1/4	1/3		
Age ± SEM	30.8 ± 3.216	28.25 ± 3.425		
EDSS ± SEM	NA	2 ± 0		
**Rapamycin sensitivity**				
Number	5	5		
Gender (M/F)	0/5	0/5		
Age ± SEM	31.2 ± 1.356	32.4 ± 1.887		
EDSS ± SEM	NA	2.2 ± 0.4062		
**AREG ELISA**				
Number		11		
Gender (M/F)		5/6		
Age ± SEM		44.82 ± 2.857		
EDSS ± SEM		1.91 ± 0.26		
**FlowSOM**				
Number	26	24	25	23
Gender (M/F)	8/18	5/19	6/19	5/18
Age ± SEM	42 ± 2.481	42.46 ± 1.910	41.92 ± 1.998	60.22 ± 1.768
EDSS ± SEM	NA	2.587 ± 0.3794	2.5 ± 0.3096	5.659 ± 0.2470
Treatment	NA	Untreated	Avonex 7/25<br>Betaferon 2/25<br>Copaxone 11/25<br>Rebif 5/25	Untreated
**Paired blood- CSF samples**				
Number		5		
Gender (M/F)		1/4		
Age ± SEM		37.8 ± 4.521		
EDSS ± SEM		2 ± 0.3162		

EDSS: expanded disability status scale; F: female; M: male; NA: non-applicable

Table S4: Primer sequences used for qPCR.

Gene	Forward primer	Reverse primer
IL-6	GAGGAGACTTGCCTGGTGAA	GCTCTGGCTTGTTCCTCACT
IL-1β	GATGAAGTGCTCCTTCCAGG	GCATCTTCCTCAGCTTGTCC
HMBS	GAATGAAGTGGACCTGGTTGT	CTGGTTCCCACCACACTCTT
TBP	TATAATCCCAAGCGGTTTGC	GCTGGAAAACCCAACTTCTG
